# Analysis of Polymer/siRNA Nanoparticle Efficacy and Biocompatibility in 3D Air–Liquid Interface Culture Compared to 2D Cell Culture

**DOI:** 10.3390/pharmaceutics17030339

**Published:** 2025-03-06

**Authors:** Sandra Noske, Martin Krueger, Alexander Ewe, Achim Aigner

**Affiliations:** 1 Rudolf-Boehm-Institute for Pharmacology and Toxicology, Clinical Pharmacology, Faculty of Medicine, Leipzig University, 04107 Leipzig, Germany; sandra.noske@medizin.uni-leipzig.de (S.N.); alexander.ewe@medizin.uni-leipzig.de (A.E.); 2 Institute of Anatomy, Leipzig University, Liebigstraße 13, 04103 Leipzig, Germany; martin.krueger@medizin.uni-leipzig.de

**Keywords:** polyethylenimine, PEI, tyrosine-modified PEI, fluoroalkyl-PEI, air–liquid interface culture, gene knockdown, RNAi

## Abstract

**Background:** Polymeric nanoparticles have been explored as efficient tools for siRNA delivery to induce RNAi-mediated gene knockdown. Chemical modifications of polyethylenimines (PEI) enhance nanoparticle efficacy and biocompatibility. Their in vivo use, however, benefits from prior analyses in relevant in vitro 3D conditions. **Methods:** We utilize a 3D ALI cell culture model for testing the biological activities and toxicities of a set of different PEI-based nanoparticles with different chemical modifications. This also includes a novel, fluoroalkyl-modified PEI. Reporter gene knockdown is directly compared to 2D cell culture. In parallel, biocompatibility is assessed by measuring cell viability and lactate dehydrogenase (LDH) release. **Results:** Knockdown efficacies in the 3D ALI model are dependent on the chemical modification and complex preparation conditions. Results only correlate in part with gene knockdown in 2D cell culture, identifying nanoparticle penetration and cellular internalization under 3D conditions as important parameters. The 3D ALI cell culture is also suitable for the quantitative determination of nanoparticle effects on cell viability and acute toxicity, with biocompatibility benefitting from PEI modifications. **Conclusions:** The 3D ALI cell model allows for a more realistic assessment of biological nanoparticle effects. A novel fluoroalkyl-modified PEI is described. Optimal preparations of PEI-based nanoparticles for siRNA delivery and gene knockdown are identified.

## 1. Introduction

Beyond enteral or parenteral application, the lung offers an interesting alternative route for drug administration, due to its high surface area, extensive capillary network with low diffusion distances, relatively low enzyme activities and the opportunity to bypass the hepatic first pass effect [1,2,3,4,5]. While these characteristics can mainly be beneficial for the systemic absorption of drug formulations after pulmonary application, some other factors show great potential for the local treatment of pulmonary diseases. The main advantages of pulmonary administration for drug delivery may include, among others and dependent on the drug, the non-invasive mode of drug application, higher bioavailability of drugs with poor gastrointestinal absorption properties and potentially reduced systemic side effects due to the much smaller dose needed for therapeutic efficacy [4,6,7,8,9]. 

Although primary human cells are the gold standard to compare in vitro results with clinical data, their limited accessibility and inter-donor variations may be problematic with regard to upscaling and reproducibility of test results [10,11]. Thus, commonly used lung epithelial cell lines offer advantages as unlimited cell sources with the possibility of long-term sub-culturing. However, especially when grown in 2D cell culture, they do not fully represent lung tissue’s in vivo properties [12,13]. The cultivation of primary or immortalized cells on a permeable tissue insert is a commonly used method to achieve more complex systems for more closely modeling the situation in the lung [14]. This air–liquid interface (ALI) setting also provides the possibility to directly expose the cells to a test agent (particle or aerosol) without its prior interaction with medium components, which could otherwise alter particle or drug characteristics [15,16]. Depending on the cell type and composition, ALI models are able to mimic healthy or diseased states of the lung (for review see [17]) or simulate different parts of the respiratory tract [11,18,19,20,21,22,23,24]. In addition to classic monocultures of pulmonary epithelial cells, different co-culture models can be used to obtain a deeper understanding of biological processes in the lung. For example, the co-culture of epithelial and immune cells can be used to analyze inflammatory responses [25,26]. Furthermore, Barron et al. analyzed the epithelial–fibroblast cross-talk in idiopathic pulmonary fibrosis using a system containing lung epithelial cells and fibroblasts [27]. A variety of commercially available ALI models provide a relative standardized platform of multicellular test systems for different parts of the respiratory tract [17]. Thus, ALI culture models allow for a still comparatively facile assessment of drug effects under relevant in vitro conditions, before going into (for example) more complicated microfluidic systems or lung slices [14,28].

The human lung adenocarcinoma cell line A549 is most commonly used for modeling the alveolar region of the lung. While different reports exist on the expression of tight junction proteins and the formation of a tight epithelial layer in A549 ALI cultures [29,30,31], the exposure to ALI conditions can be crucial for the development of characteristics closer to the in vivo situation [32]. For example, it has been shown that A549 cells displayed reduced surface tension when cultured on an air–liquid interface as compared to a liquid–liquid interface or conventional 2D cell culture. This may be caused by the secretion of a surfactant, which is typical for the alveolar space [24,33]. Furthermore, the gene expression profile of A549 cells can change dependent on the culture conditions. For example, a significant upregulation of several enzymes or proteins related to an alveolar type II cell phenotype was described after ALI exposure, including lung-specific cytochrome P450 isoenzymes and some transporters [33,34]. 

Beyond classical applications such as viral infection or toxicological studies, ALI systems can also be used as models for testing RNA therapeutics and their nanoparticle-based delivery [17,35,36]. In 2023, Lewis et al. described different lipid nanoparticles for the transfection of mRNA in Calu3 cells cultivated in an ALI system. Notably, the most promising candidate in ALI culture also showed luciferase expression in epithelial cells in vivo after intratracheal particle application [37]. Similarly, the influence of particle PEGylation on mucus permeation and small interfering RNA (siRNA) uptake was studied in ALI culture, in the context of lipid/polymer nanoparticles containing a poly(lactic-co-glycolic) acid (PLGA) core with or without polyethylenimine (PEI) and a lipid shell [38]. In another study in 16HBE cell ALI cultures, the coating of the 25 kDa PEI with Alveofact was found to lead to enhanced gene knockdown on mRNA levels compared to the core polymer, while an excess of surfactant could result in a loss of biological activity [39].

Polyethylenimines (PEIs) are widely used for nucleic acid delivery [40,41,42,43,44], but they also show molecular weight-dependent cytotoxicity [45]. These cytotoxic effects of PEI-based particles are mainly linked to their cationic and amphiphilic characteristics. By interacting with the cell membrane, PEIs can cause pore formation or membrane thinning [46]. While high cationic charge density is beneficial for nucleic acid condensation and uptake, higher charge density also results in more membrane interaction and eventually cytotoxic effects. Hence, it is important to identify the optimal ratio between nucleic acid condensation capability and cytotoxicity [47]. For example, this ratio can be improved by chemically modifying the parental PEI polymer, especially low molecular weight PEIs, to reduce toxicity [42,44,48,49]. With regard to their in vivo application, a broad biodistribution beyond the liver or the spleen was found in mice after systemic application of unmodified or chemically modified PEIs and was linked to therapeutic effects of siRNA-mediated knockdown [42,50].

While the studies described above clearly indicate the advantages of ALI culture systems for studying drug effects ex vivo, it remains still open if and to what extent these more complicated systems are needed for the assessment of biological nanoparticle properties or, in other words, to what extent classical 2D cell culture can already provide a large body of information that may be sufficient for nanoparticle characterization and selection. 

Thus, in this paper we directly compare results from 2D cell culture and 3D ALI culture. For this, we made use of an A549-EGFP/Luc reporter cell line and tested polymeric nanoparticles regarding their transfection efficacy and biocompatibility. For this, a broader set of PEI-based nanoparticles with different chemical modifications were employed, including tyrosine-grafted branched or linear low molecular weight PEIs. This modification has been described previously to improve the complex stability and biocompatibility as well as the transfection efficacy in vitro and in vivo [40,41,42,48,51], including hard-to-transfect cells [52] or solid tissue slices transfected under ALI conditions [42,53]. Their relevance for lung applications is also emphasized by the fact that these polymers allow the protection of the siRNA cargo during spray-drying and preserve the knockdown activity, as a relevant mode for pulmonary application [54]. Lipopolyplexes resulting from the combination of polymeric nanoparticles (polyplexes) and liposomes [55,56] were studied as well. Furthermore, we present and include a novel fluoroalkyl-modified 10 kDa branched PEI, since fluorocarbon modification has been described to decrease the interaction of the particle with serum components, and thus to reduce its aggregation in biological media. This could be helpful to overcome the mucus barrier in the respiratory tract [57,58].

## 2. Materials and Methods

### 2.1. Cell Culture

All cell culture plastics and consumables were purchased from Sarstedt (Nümbrecht, Germany). The culture medium (IMDM) as well as the alanyl-glutamine supplement were from Sigma Aldrich (Taufkirchen, Germany), while fetal calf serum was purchased from Serana (Pessin, Germany). The cell lines A549 (human alveolar cell carcinoma) and H441 (human lung adenocarcinoma) were obtained from ATCC/LGC Promochem (Wesel, Germany) and the respective sub-cell lines stably expressing the reporter gene *EGFP/Luc* or *Luc*, respectively, were established by stable lentiviral transduction as described previously [41]. Independent of the cell culture system (2D or 3D ALI), the cells were cultivated in Iscove’s Modified Dulbecco Medium (IMDM) supplemented with 10% FCS and 2 mM alanyl-glutamine at 37 °C and 5% CO_2_ under saturated humidity conditions and sub-cultured when a confluency of 70–80% was reached.

### 2.2. Air–Liquid Interface Culture

For establishing the ALI culture, 35,000 A549-EGFP/Luc cells in 300 µL medium were seeded onto a 24-well insert (apical side). The compartment below the insert (basal compartment) was filled with 500 µL medium as well. The cells were cultivated for 2 days to reach confluency. Then, the apical medium was removed to create the ALI conditions. The cells were further cultivated for 2 days before the system was used for transfection. A medium exchange was performed every other day after seeding.

### 2.3. Fluoroalkyl-PEI Synthesis, Complex Preparation and Transfection

The tyrosine modification of linear and branched 10 kDa PEI (LP10, P10), purchased from Sigma Aldrich (Taufkirchen, Germany) and Polysciences (Eppelheim, Germany), was performed as described previously [40,42]. Glycidyl 2,2,3,3,4,4,5,5-octafluoropentyl ether was obtained from Sigma Aldrich (Taufkirchen, Germany), triethylamine (NEt_3_) and ethanol from Carl Roth (Karlsruhe, Germany), dialysis tube 3.5 kDa MWCO and regenerated cellulose (RC) from Serva (Heidelberg, Germany) and CDCl_3_ from Deutero (Kastellaun, Germany).

Three fluoroalkyl-PEI derivatives were prepared, with 50%, 25% and 5% of the PEI monomers being modified. Branched 10 kDa PEI (100 mg, 2.32 mmol ethylenimine (EI) monomer) was dissolved in 3 mL ethanol in a dram vial and 1 equivalent of NEt_3_ to the corresponding amount of fluoroalkyl was added. Under stirring, the glycidyl 2,2,3,3,4,4,5,5-octafluoropentyl ether at different molar ratios was dropped into the polymer solution. In detail: for 50%, 0.6 eq of EI, 1.4 mmol, 401 mg, 250 µL; for 25%, 0.3 eq of EI, 0.7 mmol, 200 mg, 126 µL and for 5%, 0.06 eq of EI, 0.14 mmol, 40.1 mg, 25 µL. The mixtures were stirred for 48 h at room temperature. The polymers were purified by dialysis (RC, MWCO 3.5 kDa) against ethanol for 2 days. After the solvents were removed in vacuo, the fluoroalkyl-modified PEIs were obtained as white viscous gels. 

The percentage of fluoroalkyl per PEI monomer was determined via ^1^H NMR (Avance III 400 MHz, Bruker Corp., Billerica, MA, USA) by comparing integrals of the ethylenimines to the methine proton “1” of the fluoroalkyl group (see Figure S1C–E). To this end, polymers were dissolved at a final concentration of 20 mg/mL in CDCl_3_ and spectra were recorded at room temperature. The chemical shifts were reported as values in ppm relative to the CDCl_3_ signal. Spectra were analyzed using the MNova software V14.1 (Mestrelab Research, Santiago de Compostela, Spain). For calculation, the ratio of the integrals between the ethylenimines (2.89–2.34 ppm) and the methine proton “1” (~6.3–5.9 ppm) were compared (see Figure S1C–E). 

P10F_50_ ^1^H NMR (400 MHz, CDCl_3_) σ: 6.3–5.9 (m, ^1^H, CF_2_H “1”), 4.05–3.43 (m, 4.74 H, -CF_2_CH_2_-O (“2”), O-CH_2_-CH-OH- (“3”), -CH_2_-CH-OH-CH_2_- (“4”), 2.89–2.34 (m, 8.2 H, PEI -NH-CH_2_-CH_2_-, NH-CH_2_-CH-OH-(“5”).

P10F_25_ ^1^H NMR (400 MHz, CDCl_3_) σ: 6.33–5.89 (m, ^1^H, CF_2_H “1”), 4.12–3.49 (m, 5.24 H, -CF_2_CH_2_-O (“2”), O-CH_2_-CH-OH- (“3”), -CH_2_-CH-OH-CH_2_- (“4”), 2.89–2.34 (m, 16.55 H, PEI -NH-CH_2_-CH_2_-, NH-CH_2_-CH-OH-(“5”).

P10F_5_ ^1^H NMR (400 MHz, CDCl_3_) σ: 6.28–6.02 (m, 1.25 H, CF_2_H “1”), 4.04–3.58 (m, 5.53 H, -CF_2_CH_2_-O (“2”), O-CH_2_-CH-OH- (“3”), -CH_2_-CH-OH-CH_2_- (“4”), 2.87–2.38 (m, 80.68 H, PEI -NH-CH_2_-CH_2_-, NH-CH_2_-CH-OH-(“5”).

The biological activity for the three fluoroalkyl-modified PEIs (“P10F”) was first tested in H441-Luc reporter cells at various polymer/siRNA mass ratios, similar to that described below. The results are shown in Figure S2. For the subsequent experiments in this study, the 25% modified PEI (P10F-25%) was selected and referred as “P10F” throughout the manuscript. 

The different polymers were complexed with siRNA at the previously determined optimal mass ratios, i.e., mass ratio 2.5 in the case of the chemically modified polymers and mass ratio 7.5 in the case of the unmodified P10. Given the differences in molecular weight of the polymers, this mass ratio translates into different N/P ratios (Supplementary Materials Table S1). For clarity, however, mass ratios are given in the text. The siRNAs used in this study are listed in the Supplementary Materials Table S2. The complexation was either performed in the standard complexation buffer for use in cell culture experiments (150 mM NaCl, 10 mM Hepes, pH 7.4; HN buffer) or in 10% trehalose buffer containing 20 mM Hepes, pH 7.4. For complexation of 1 µg nucleic acid, the siRNA and the polymer were separately diluted in 31.25 µL complexation buffer each, prior adding the siRNA dilution to the polymer dilution. In the later experiments, the complexation of the LP10Y/siRNA complexes was performed by diluting 1 µg nucleic acid and polymer in only 5 µL, respectively, in order to reduce the total complexation volume. After mixing, the complexes were allowed to form for 30 min at room temperature. For liposome preparation, 5 mg DPPC (Avanti Polar Lipids, Alabaster, AL, USA) was dissolved in chloroform/methanol (2:1, *v*/*v*) and mixed in a 5 mL round bottom flask. The solvent evaporation was performed at 55 °C using a rotary evaporator and a programmable vacuum pump (30 s 800 mbar, 5 min 500 mbar, 30 min 2 mbar, 60 min 2 mbar). The thin lipid film was hydrated using 10% trehalose containing 20 mM Hepes and further incubated for 5 min at 55 °C in an ultrasound bath sonicator. Subsequently, the liposome solution was extruded 21 times through a 200 nm polycarbonate membrane using a preheated Mini-Extruder (Avanti Polar Lipids, Alabaster, AL, USA). The lipopolyplex (LPP) formation was performed by the addition of DPPC liposomes after complexation, at a mass ratio of 10 based on the polymer amount. Equal volumes of the required DPPC amount and the complex were vigorously mixed by vortexing. After 5 min treatment in an ultrasonic bath, the LPPs were allowed to sit for 90 min at room temperature. Prior to transfection, the particles prepared at the lower complexation volume of 10 µL were diluted in order to reach the same 62.5 µL volume.

### 2.4. Particle Uptake After Transfection of ALI Culture

Prior to transfection, the ALI culture was established as described above. On the day of the transfection, the medium in the basal compartment was replaced and 300 µL medium was added to the apical side of the ALI culture. The systems were transfected with complexes containing 1 µg or 2 µg (75 pmol or 150 pmol, respectively) of a HiLyte647-labled siRNA added to the apical medium for 8 h. After aspirating the apical medium to restore ALI conditions, the system was further incubated. The siRNA uptake was analyzed by flow cytometry 24 h after transfection. The uptake efficacy was determined as the increase in mean HiLyte647 signal relative to the untransfected control.

### 2.5. Reporter Gene Knockdown After siRNA Transfection

For the 2D cell culture experiments, reporter cells were seeded in 24-well plates at a density of 35,000 cells per 500 µL. Unless stated otherwise, no medium exchange was performed prior to transfection. Then, 24 h after seeding, the cells were transfected with nanoparticles containing a target-specific siRNA (Luciferase: siLuc3, EGFP: siEGFP) or a negative control siRNA (siLuc2, with Luc2 not being expressed in the cells and the siRNAs thus targeting an irrelevant gene). After transfection with 0.4 µg siRNA (30 pmol; corresponding to a concentration of 54–57 nM, depending on the complexation volume), the cells were incubated for 96 h (A549-EGFP/Luc cells) or 72 h (H441-Luc cells). The luciferase activity was finally determined using a luminometer, whereas the EGFP intensity was analyzed by flow cytometry. The knockdown efficacy after siLuc3 transfection was defined as the decrease in relative luminescence units per second (RLU/s) relative to the corresponding siCtrl transfection. The knockdown after siEGFP transfection was defined as the decrease in the median EGFP intensity relative to the respective siCtrl transfection.

For the transfection of the ALI culture, the ALI system was established as described above. Prior to transfection, the medium in the basal compartment was replaced with 500 µL fresh medium and 300 µL medium was added to the apical compartment. Unless stated otherwise, nanoparticles containing 2 µg siRNA (150 pmol; corresponding to a concentration of 270–295 nM, depending on the complexation volume) were added to the apical compartment and incubated for 8 h. The cells were transfected with an siRNA targeting EGFP (siEGFP) or a negative control siRNA (siCtrl; siLuc2). After 8 h, the apical medium was aspirated for restoring the ALI conditions. Furthermore, the medium in the basal compartment was exchanged every other day. The green fluorescence signal was monitored at 96 h after transfection using a Keyence BZ-X810 (Keyence, Neu-Isenburg, Germany) and finally analyzed by flow cytometry. The knockdown efficacy after siEGFP transfection was determined as the decrease in the median EGFP signal relative to the corresponding siCtrl transfection.

### 2.6. Luciferase Assay

The luciferase activity was analyzed 96 h (A549-EGFP/Luc) or 72 h (H441-luc) after transfection of the cells in 2D cell culture. For this, the medium was discarded, and the cells were lysed by adding 200 µL (A549-EGFP/Luc cells) or 300 µL (H441-Luc cells) Luciferase Cell Culture Lysis Reagent (Promega, Mannheim, Germany) per well, prior to incubation for 30 min at room temperature on a shaker. For luminescence measurement, 10 µL of the cell lysate was mixed with 25 µL luciferin reagent (Beetle-Juice Kit, PJK, Kleinblittersdorf, Germany) in a test tube and immediately measured in a luminometer (Berthold, Bad Wildbad, Germany).

### 2.7. Flow Cytometry

The siRNA uptake was analyzed 24 h after transfection of the ALI system. Prior to trypsinization, the medium in the basal compartment was removed and the membrane was washed twice with pre-warmed PBS. After detaching from the culture inserts by trypsinization, the cells were collected by centrifugation at 3000 rpm for 5 min. The cell pellet was suspended in 500 µL FACS buffer (PBS, 1% FCS, 0.1% NaN_3_). A minimum of 10,000 events in the single cell gate derived from viable cell population were measured using an Attune^®^ Acoustic Focusing Cytometer (638 nm laser with 660/20 nm filter configuration) and analyzed in the manufacturer’s software version 2.1 (Applied Biosystems, Foster City, CA, USA).

The EGFP expression levels were analyzed 96 h after transfection of the 2D cell culture or ALI culture. After harvesting the cells, the pellet was suspended in 500 µL FACS buffer (PBS, 1% FCS, 0.1% NaN_3_). A minimum of 10,000 events in the single cell gate derived from the viable cell population were measured using an Attune^®^ Acoustic Focusing Cytometer (488 nm laser with 530/30 nm filter configuration) and analyzed in the manufacturer’s software version 2.1 (Applied Biosystems, Foster City, CA, USA).

### 2.8. Acute Toxicity (LDH Release)

The acute cell damage upon transfection of the ALI culture was determined by measuring lactate dehydrogenase (LDH) release into the culture medium, using the Cytotoxicity Detection Kit from Roche (Mannheim, Germany) according to the manufacturer’s protocol. The ALI culture was established as described above, and after a medium exchange in the basal compartment, the system was transfected with nanoparticles containing 2 µg siRNA in 300 µL medium in the apical compartment. After the 8 h transfection period, medium samples (technical duplicates) from the basal and the apical compartment were collected, and the ALI conditions were restored. The maximum LDH release for each time of analysis was determined by the total lysis of the cells with Triton X-100 at a final concentration of 2% (positive control) for 30 min at 37 °C, while the basal LDH release was determined from untransfected ALI cultures as the negative control and freshly prepared medium was used for background signal subtraction. In a 96-well plate, 50 µL reagent mix was incubated with 50 µL medium sample for 30 min at room temperature in the dark. After stopping the reaction by adding 50 µL 1 M acetic acid, the absorbance at 450 nm and 620 nm as reference wave length was measured in a plate reader (Thermo Fisher, Schwerte, Germany). Acute cell damage was calculated as the LDH release relative to the positive control (pos-ctrl = 1) after background subtraction.

### 2.9. Cell Viability

The cell viability/metabolic activity of ALI cultures was analyzed 96 h after transfection using the colorimetric WST-8 Cell Counting Kit (Dojindo MolecularTechnologies EU, Munich, Germany). The ALI system was established and transfected with 2 µg siRNA, as described above. After 4 days of incubation, the medium from the basal compartment was removed and the insert was washed twice with pre-warmed PBS. Then, 400 µL of a 1:10 dilution of the WST-8 reagent in serum-free medium was added to the apical compartment and the cells were incubated for 30 min at 37 °C. For measuring the enzyme-mediated formazan formation from the WST-8, duplicates of 50 µL per sample of the reagent-medium mix were transferred into a 96-well plate, and the absorbance at 450 nm (with 620 nm as reference wavelength) was measured in a plate reader (Thermo Fisher, Schwerte, Germany). The reagent–medium mix incubated at 37 °C without cells was measured and subtracted for background signal correction. The cell viability was given as the absorption value relative to the untreated control (UT = 1) after background subtraction.

### 2.10. Particle Characterization

The hydrodynamic diameters and ζ-potentials of the (lipo-)polyplexes were determined by dynamic light scattering (DLS) and phase analysis light scattering (PALS), using the Zeta sizer Advanced Series Ultra Red (Malvern Panalytical, Kassel, Germany). For particle formation, 2.5 µg siRNA was complexed in a final volume of 125 µL complexation buffer. In the case of (DPPC)-LP10Y/siRNA particles with the lower complexation volume, the particle formation was performed as described above and the volume was adjusted to 125 µL only after the complex formation. The nanoparticles were diluted in 1 mL Millipore water and the measurements were performed at 25 °C using the refractive index and viscosity of water in the analysis and employing the manufacturer’s software. For hydrodynamic diameter calculation, three measurements with an automatic measurement process (a minimum of 15 runs) were performed at an angle of 90° and the multiple narrow mode was used for data processing. The hydrodynamic diameters were displayed as the mean of three independent measurements with standard deviation. The ζ-potentials were measured in three separate runs (a maximum of 40 sub-runs) and the monomodal analysis model was used for data processing, resulting in the mean ζ-potential and standard deviation.

The influence of the culture medium on the hydrodynamic diameter and ζ-potential of the particles was determined by Zeta Sizer measurement after incubation with medium containing FCS for 3 h and 24 h. Complexes containing 2.5 µg siRNA were assembled in 125 µL as described above. The particles were mixed with 500 µL full medium, previously filtered to minimize large aggregates in the medium background, and incubated for 3 h or 24 h. For measurement, 200 µL of the particle–medium mix was diluted with 1 mL Millipore water and further processed as described above. 

For further analysis of particle size and shape, the particles were imaged using transmission electron microscopy (TEM). The complex preparation was performed as described for the Zeta Sizer measurement and subsequently, droplets of 5 µL were placed on formvar-coated copper grids for 60 s. After that, the fluid was removed with filter paper, followed by air drying. Next, the samples were stained with 1% uranyl acetate (Serva, Heidelberg, Germany) in H_2_O for 2 min. After rinsing in H_2_O and consecutive air drying, the samples were analyzed with a Zeiss SIGMA electron microscope (Zeiss, Oberkochen, Germany) equipped with a STEM detector. Images were taken at 20,000× magnification and the particle sizes were analyzed using ImageJ version 1.4.3.67.

### 2.11. Statistics

The results of at least two transfection experiments are presented as the mean ± standard error of the mean (SEM) or standard deviation (SD). Data analysis was performed using linear regression for correlation analysis and Welch’s *t*-test to determine significance using SigmaPlot software (version 14.0, Systat Software, Düsseldorf, Germany), with *p*-values < 0.03 indicating significant differences between the control and specific transfection group. Significance is indicated by *, *p* < 0.03; **, *p* < 0.01 and ***, *p* < 0.001.

## 3. Results

### 3.1. Modified PEIs Show Enhanced Knockdown Efficacy in 2D Cell Culture, Dependent on the Complexation Conditions

In A549 cells, a set of different PEI-based nanoparticles were first tested for their knockdown efficacies in 2D cell culture. These included polyplexes based on tyrosine-modified 10 kDa branched or linear PEI (P10Y and LP10Y, respectively) described previously [40,42] and their corresponding DPPC lipopolyplex derivatives [55,56] as well as a novel, fluoroalkyl-modified 10 kDa PEI (P10F). The P10F polymers were synthesized according to the synthesis scheme shown in Figure S1A and as described in the Materials and Methods. By modulating the stoichiometry, three polymers with different degrees of fluoroalkyl modification were obtained (Figure S1B). ^1^H-NMR analysis revealed the correct products (polymer (2) in the above synthesis scheme with the different degrees of fluoroalkyl modification), as identified by the characteristic peaks (Supplementary Materials Figure S1C–E; see also Materials and Methods, Section 2.3 for details). 

**Figure 1:**
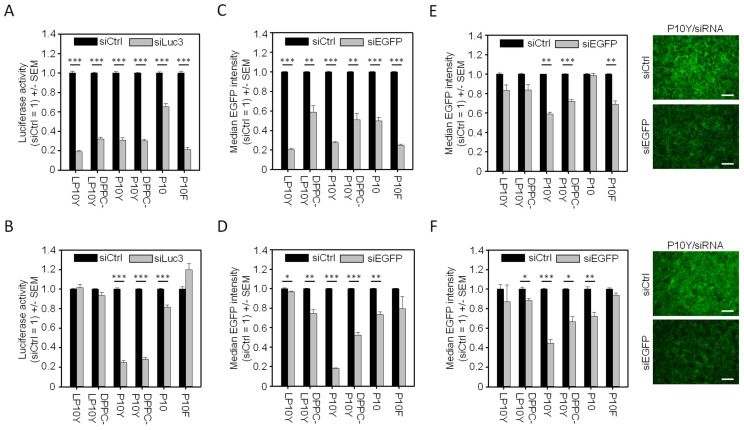
Reporter gene knockdown in A549-EGFP/Luc cells 96 h after transfection with nanoparticles prepared in (**A**,**C**,**E**) HN buffer or (**B**,**D**,**F**) 10% trehalose buffer. Luciferase (**A**,**B**) and EGFP knockdown (**C**,**D**) results from 2D cell culture in comparison to EGFP knockdown in 3D ALI culture (**E**,**F**). Rightmost panels: representative original fluorescence images after transfection with P10Y/siRNA nanoparticles for illustrating the decrease in EGFP expression (scale bar: 500 μm). Data are reported as mean ± SEM. Significance indicated by * = *p* < 0.03; ** = *p* < 0.01; *** = *p* < 0.001 between negative control and specific siRNA transfection.

When prepared in HN buffer for complexation at the previously defined optimal polymer/siRNA mass ratio of 2.5 (see Supplementary Materials Table S1 for corresponding N/P ratios), all chemically modified PEIs led to nanoparticles with higher knockdown efficacies (~70–80% kd) as compared to the non-modified 10 kDa PEI (P10; ~35% kd). Here and elsewhere, knockdown was determined by the difference in luciferase expression between luciferase reporter cells transfected with negative control (siCtrl) vs. luciferase-specific siRNA (siLuc3) at 96 h after transfection (Figure 1A). Particularly high efficacies were also seen in the case of two of the three novel P10F polymers (P10F_50_ and P10F_25_) largely independent of the polymer/siRNA mass ratio (2.5, 3.75 or 5.0), with the only exception being the low-degree (5%) fluoroalkyl-modified P10F_5_ (Figure S2). Thus, the latter was not pursued further, and P10F_25_ was selected for subsequent experiments (hereinafter referred to as P10F and employed at mass ratio of 2.5). 

When switching to 10% trehalose buffer for complex formation, however, P10F-based polyplexes as well as the polyplexes/lipopolyplexes based on the linear, tyrosine-modified PEI LP10Y completely lost their activity (Figure 1B). In contrast, no loss of knockdown efficacy was observed in the case of the branched P10Y-based polyplexes and lipopolyplexes. The analysis of nanoparticle hydrodynamic diameters and ζ-potentials revealed overall smaller particles in the case of the LP10Y-based polyplexes when compared to their P10Y-based counterparts (Table 1). Furthermore, significant differences in ζ-potentials were detected between particles of the branched (around −10 mV) and linear tyrosine-grafted PEIs (25–30 mV). The switch from HN buffer to 10% trehalose buffer for complexation considerably affected the hydrodynamic diameters of the nanoparticles based on linear tyrosine-grafted PEI, but not of the nanoparticles based on P10Y, where no significant change in particle hydrodynamic diameter was seen. On the other hand, the abolishment of transfection efficacy of the P10F/siRNA complex seems not to be triggered by a change in particle hydrodynamic diameter, since again no significant differences in particle hydrodynamic diameters were found. Differences in ζ-potential cannot explain the differences in biological activity either, since no correlation between ζ-potential and knockdown efficacy was seen for any of the nanoparticles (Table 1).

**Figure 2:**
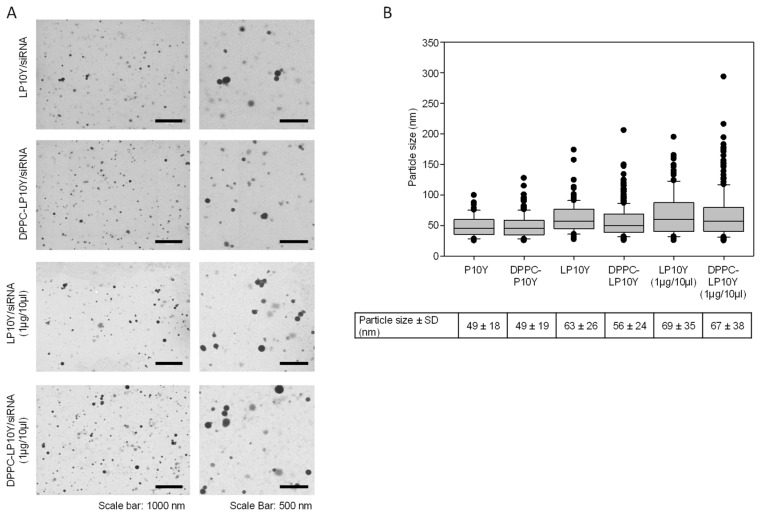
(**A**) Transmission electron microscopy (TEM) pictures of different complexes at two magnifications. (**B**) Quantitation of nanoparticle sizes, as determined by TEM.

Nanoparticles prepared in the trehalose buffer were also analyzed by transmission electron microscopy (TEM), which revealed largely round-shaped particles (Figure 2A) with mean sizes below the Zeta Sizer-based measurements of hydrodynamic diameters (Figure 2B). However, some larger nanoparticles in the size range of several hundred nanometers were found. Some differences between the polymers and the concentrations for complexation were again observed, with polyplexes or lipoplyplexes prepared in the lower complexation volume exhibiting larger sizes, as seen before (Table 1). 

Knockdown results largely similar to the luciferase experiments were obtained when analyzing EGFP as a reporter gene. Again, high knockdown efficacies were seen in the case of the LP10Y-, P10Y- and P10F-based polyplexes, while P10-based polyplexes and the lipopolyplexes were less efficient (Figure 1C). Switching to trehalose buffer led again to the reduction or loss of gene knockdown dependent on the polymer, with P10Y-based polyplexes and lipopolyplexes fully retaining their activity (Figure 1D). This also demonstrates that the determination of knockdown efficacies was largely independent of the target gene. Still, it has to be kept in mind that both luciferase and EGFP reporter genes show aberrantly high expression levels due to their ectopic overexpression, which may well lead to somewhat lower knockdown efficacies. Thus, results from reporter cell lines may rather underestimate the biological activities of siRNA-containing nanoparticles.

### 3.2. Knockdown in 3D ALI Culture Does Not Correspond to 2D Efficacies 

For assessing knockdown efficacies in a more relevant 3D system, we switched to an air–liquid interface (ALI) culture model of the same A549 (double) reporter cell line. As to be expected, the analysis of EGFP levels upon transfection with specific (siEGFP) vs. negative control siRNA (siCtrl) revealed overall lesser knockdown efficacies of the nanoparticles prepared in HN buffer for complexation, when compared to 2D cell culture (Figure 1E). Polyplexes and lipopolyplexes based on chemically modified branched PEIs showed somewhat higher gene knockdown than their linear counterparts, while the non-modified P10 produced inactive polyplexes. However, P10 (and to a lesser extent its tyrosine-modified polyplexes) benefitted from switching to 10% trehalose buffer for complexation (Figure 1F). This improvement was not seen in the case of their linear counterparts. In contrast, P10F-based complexes completely lost their biological activity when prepared under these conditions. Again, the comparison with physicochemical (lipo-)polyplex properties does not solely indicate the nanoparticle hydrodynamic diameter or ζ-potential as the single major underlying reason for the observed differences in 3D ALI culture.

**Figure 3:**
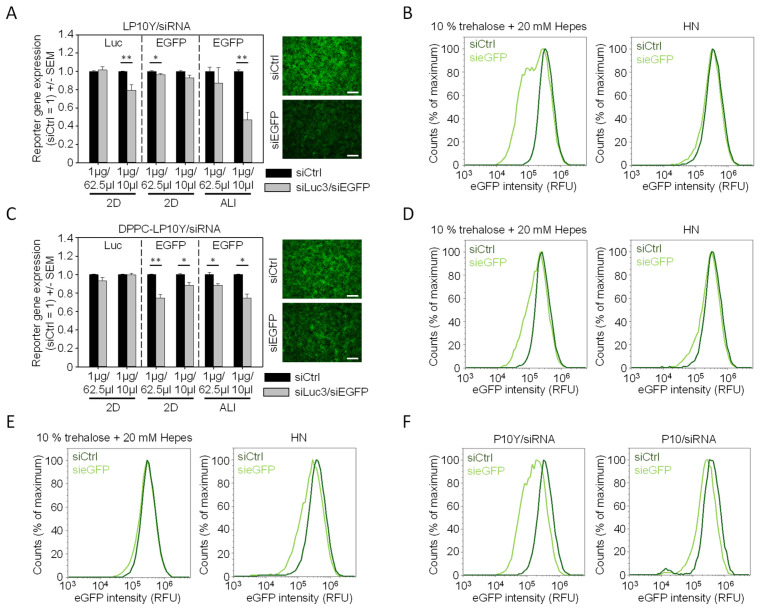
Optimization of knockdown efficacies for (**A**) LP10Y/siRNA polyplexes and (**C**) LP10Y/siRNA lipopolyplexes by decreasing the complexation volume. Right: original fluorescence images for illustrating EGFP knockdown in A549-EGFP/Luc cells (scale bar: 500 μm). Corresponding original flow cytometry histograms for (**B**) LP10Y/siRNA polyplexes and (**D**) LP10Y/siRNA lipopolyplexes, prepared under buffer conditions as indicated. (**E**) Results from P10F/siRNA complexes. (**F**) Direct comparison between P10Y/siRNA and P10/siRNA complexes, prepared in 10% trehalose buffer. Data are reported as mean ± SEM. Significance indicated by * = *p* < 0.03; ** = *p* < 0.01 between negative control and specific siRNA transfection.

Considering the somewhat surprising loss of 2D knockdown efficacies of LP10Y-based nanoparticles when prepared in 10% trehalose buffer instead of HN buffer, we further analyzed the modification of complexation conditions by considerably reducing the complexation volume from 62.5 µL to 10 µL. In the case of LP10Y/siRNA polyplexes, this led to a doubling of complex hydrodynamic diameters that was not seen for the already larger DPPC-LP10Y/siRNA lipopolyplexes (Table 1). The ζ-potential was largely unaffected. On the biological side, this approach of volume reduction led to a slight gain in luciferase knockdown in the case of LP10Y/siRNA complexes in 2D cell culture (Figure 3A, left), while no benefit was observed for EGFP knockdown (Figure 3A, center) or when analyzing lipopolyplexes (Figure 3C, left and center). In contrast, in 3D ALI culture the knockdown efficacy of LP10Y/siRNA complexes was substantially increased to >50% upon their preparation in smaller volumes, and to a smaller extent, this effect was also seen for lipopolyplexes (Figure 3A,C, right bars). Interestingly, this indicates that larger nanoparticle hydrodynamic diameters seem to improve knockdown efficacies in 3D ALI culture.

Thus, complex activities depend on the chemical polymer modification as well as the conditions for complexation, and the discrepancies between 2D and 3D knockdown efficacy may be in part, but not solely explained by differences in complex hydrodynamic diameters and ζ-potentials. Our results also identify P10Y/siRNA complexes prepared in 10% trehalose buffer—as well as LP10Y/siRNA complexes prepared in 10% trehalose buffer and at smaller volumes—as being the most efficient for gene knockdown under 3D ALI conditions. A dose dependency was observed as well, with half of the amounts of LP10Y/siRNA or P10Y/siRNA complexes leading to a slight decrease in knockdown efficacy (Figure S3). On the other hand, a further increase in siRNA amounts would have led to severe toxicity, at least in the case of the unmodified 10 kDa PEI. Thus, 2 µg siRNA was defined as the standard amount in the subsequent experiments. 

The underlying mechanisms and parameters affecting knockdown efficacies may include nanoparticle stability in biological media and cellular uptake. Indeed, the direct comparison between nanoparticles based on LP10Y vs. P10Y revealed major differences in uptake efficacy, as seen by substantially higher signals in flow cytometry in the case of the P10Y-mediated delivery of a fluorophore-labeled siRNA (Figure S4A,B). This, however, was not mirrored by differences in gene knockdown (Figure S4C). In fact, while a dose dependence was observed in both processes, the direct comparison of gene knockdown revealed identical efficacies (Figure S4C). The activity of the nanoparticles in serum-containing medium already indicated their stability in biological media. Still, we analyzed the hydrodynamic diameters upon prolonged incubation in serum-containing medium for up to 24 h. As shown in Figure S4D, after a slight hydrodynamic diameter increase within the first 3 h, no further alterations in hydrodynamic diameters were observed, indicating the absence of aggregation or decomposition. The only exceptions were DPPC-P10Y lipopolyplex-based nanoparticles, which were already larger at the beginning and showed an increase in their initial hydrodynamic diameter. However, we conclude from these data that nanoparticle uptake and stability do not represent major determinants for explaining the differences observed in our 3D ALI model. 

### 3.3. Closer Analyses Reveal Nanoparticle-Dependent Differences in the Cellular Distribution of Gene Knockdown Efficacies in 3D ALI Culture

Considering the differences observed in 3D ALI culture with regard to knockdown efficacies of nanoparticles prepared in HN buffer vs. 10% trehalose buffer, we further analyzed the distribution of the EGFP knockdown on the single cell level. LP10Y/siRNA complexes prepared in HN buffer showed almost identical histograms with only a very minor cell population shifting to the left (Figure 3B, right), corresponding to the little gene knockdown shown in Figure 1E (leftmost bars). In stark contrast, in the case of the same complexes prepared in 10% trehalose buffer under optimized conditions (Figure 3A, rightmost bars), a major percentage of cells showed a substantial shift towards lower EGFP expression (Figure 3B, left). 

Notably, EGFP levels in other cells remained essentially unchanged (note the unaltered right flank of the histogram peak), demonstrating that not all cells were equally hit by the nanoparticle-mediated gene knockdown under 3D conditions. This also indicates that bar diagrams giving the median EGFP levels do not fully reveal this broader cell distribution. A similar trend was seen in the case of the DPPC-LP10Y/siRNA lipopolyplexes, although with a slightly higher efficacy when prepared in HN buffer as well as a smaller percentage of cells with gene knockdown upon lipopolyplex preparation in 10% trehalose buffer (Figure 3D). In the case of P10F-based complexes prepared in HN buffer, this distinction between transfected and non-transfected cells was not seen; rather, the histogram showed a more uniform shift of the whole peak to left (Figure 3E, right). In line with the bar diagram in Figure 1F (rightmost bars), P10F/siRNA showed essentially no activity when prepared in 10% trehalose buffer. Thus, while complexation in 10% trehalose buffer usually increased nanoparticle knockdown efficacies in 3D ALI culture over their counterparts prepared in HN buffer, these complexes showed an opposite trend, leading to the complete abolishment of bioactivity (Figure 3E, left). Similar to P10F/siRNA polyplexes prepared in HN buffer, the complexation of siRNA in the parent 10 kDa PEI generated nanoparticles that led to a general peak shift to the left, indicating a more uniform, albeit not very profound EGFP knockdown in all cells (Figure 3F, right). The tyrosine modification of this PEI, resulting in the P10Y polymers, profoundly enhanced knockdown efficacies (compare Figure 1F). As demonstrated in Figure 3F, left, this was not due to a generally more pronounced left-shift of all cells in the histogram plot. Rather, while the right flank of the peak remained very similar, a major cell population showed a more profound reduction in EGFP intensity (left flank of the peak), indicating that in these cells, the nanoparticles were more efficient in the induction of a gene knockdown. 

These results thus demonstrate that 3D ALI cultures are able to delineate nanoparticle-dependent differences with regard to the extent and cellular distribution of gene knockdown. P10Y/siRNA and LP10Y/siRNA nanoparticles prepared in 10% trehalose buffer are most efficient, with some differences in knockdown results regarding the percentage of cells and the degree of EGFP downregulation. 

### 3.4. Weakly Positive Correlation Between 2D and 3D Gene Knockdown

**Figure 4:**
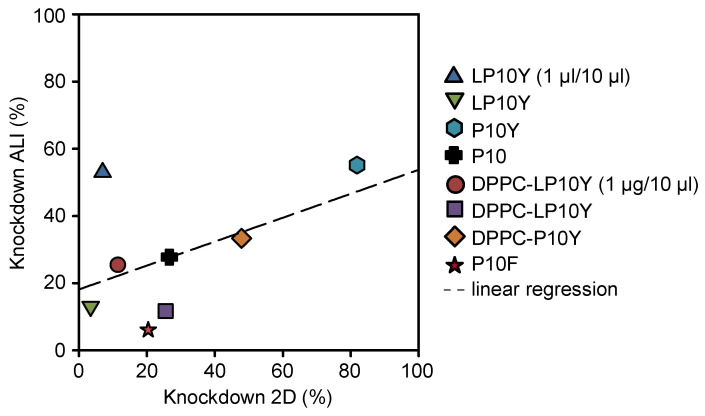
Correlation between knockdown efficacies of polyplexes/lipopolyplexes after transfection of A549-EGFP/Luc cells, cultivated in 2D cell culture vs. ALI culture. Results from linear regression are given (R^2^ = 0.25).

Bearing in mind our results on the differences in knockdown efficacies of the different nanoparticles between 2D cell culture and 3D ALI culture, we directly compared the percentages of gene knockdown in a correlation plot. As shown in Figure 4, a positive correlation was determined, with the R value of a linear regression analysis revealing a weak positive linear correlation (R^2^ = 0.25). Thus, the 2D cell culture does not fully represent efficacies in the 3D. The deviation of some nanoparticles from an overall correlation, which cannot be easily predicted from other nanoparticle properties, again underlines the necessity for testing beyond the standard 2D cell culture conditions. This applies, for example, to the LP10Y/siRNA particles complexed in a smaller volume, which showed a significant improvement in biological activity in the more complex system.

### 3.5. Assessment of Nanoparticle Biocompatibility in 3D ALI Culture

**Figure 5:**
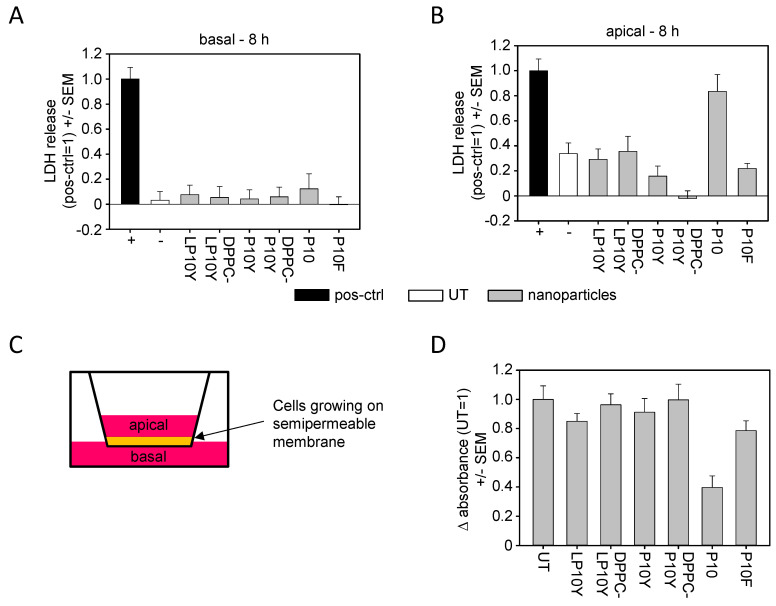
Assessment of acute cell damage after transfection of A549-EGFP/Luc, as measured by LDH release into the lower (**A**) or upper (**B**) medium reservoir. (**C**) Scheme defining the upper (apical) and lower (basal) compartment. (**D**) Long-term effects on cell viability, as determined by cellular dehydrogenase activity after 96 h. Data are reported as mean ± SEM.

Beyond biological efficacy as indicated by gene knockdown, high biocompatibility is another major criterion in successful nanoparticle development. In terms of nanoparticle toxicity, acute cell/membrane-damaging nanoparticle properties, as seen by measuring LDH release, can be distinguished from more subtle cytotoxic effects affecting cell viability over time. In terms of short-term effects, our 3D ALI culture setup also allowed for distinguishing between the induction of membrane damage upon immediate exposure to the nanoparticle-containing solution, as to be seen by LDH release into the upper (‘apical’) compartment of the well, and the LDH release into the lower (‘basal’) compartment requiring prior nanoparticle penetration into the deeper cell layers (Figure 5). 

As to be expected when considering this penetration barrier, little LDH release was observed into the basal compartment at 8 h after application of the nanoparticle solution onto the apical side (Figure 5A). Still, nanoparticles based on the non-modified 10 kDa PEI (P10) showed somewhat higher values, which is in line with previous findings demonstrating the beneficial effects of chemical PEI modifications on nanoparticle biocompatibility. When measuring LDH levels in the apical compartment at the same time point, considerably more profound effects were observed, especially with regard to the direct cell-damaging effects of the chemically unmodified P10/siRNA nanoparticles (Figure 5B,C).

Since the ALI culture requires the removal of the apical, nanoparticle-containing solution after 8 h in order to restore the ALI conditions, this prevented the measurement of LDH release at later time points, and the determination of LDH release into the basal compartment yielded no results in terms of LDH level increase. Thus, longer-term cytotoxic effects would rather have to rely on the determination of cell viability. 

The WST-8 assay is based on the metabolism of the water-soluble tetrazolium salt WST-8 into its corresponding formazan by a mitochondrial dehydrogenase, with the enzyme activity being proportional to the number of viable cells. While usually performed in a 96-well plate format, our results demonstrate that the 3D ALI setting also allowed for the direct addition of the WST-8 reagent into the apical well and a subsequent incubation. 

Indeed, when the WST-8 reagent was added at day 4 after exposure to the nanoparticles and incubated for 30 min prior to harvesting the solution and determining its formazan concentration by absorption measurements, differences in cell viability could be accurately detected (Figure 5D). Again, the non-modified P10/siRNA complexes showed comparatively poor biocompatibility, with a > 50% reduction in cell viability. P10F-based complexes and, to a lesser extent, LP10Y/siRNA complexes exhibited some impairment of cell viability as well, while the other nanoparticles, especially the lipopolyplexes, did not lead to any changes over untreated wells. 

Thus, the 3D ALI culture setting allows us to determine the acute short-term cell-damaging effects of nanoparticles, as well as their longer-term effects on cell viability. Tyrosine-modified nanoparticles, in particular P10Y-based polyplexes or lipopolyplexes, showed the highest biocompatibility.

## 4. Discussion

The pulmonary application of siRNA may offer several advantages over established therapies; however, this strategy meets major hurdles, especially with regard to siRNA delivery. The administration of naked siRNAs has been tested in mouse models for the treatment of different diseases, including tuberculosis [59], idiopathic pulmonary fibrosis [60], asthma [61] or lung cancer [62]; for further review, see [63,64]. Still, beyond siRNA deposition in the desired region of the respiratory tract, other issues and barriers like mucus, surfactant, siRNA penetration in the epithelial tissue as well as efficient cellular internalization, intracellular release of the siRNA and endosomal escape need to be considered as well. Nanoparticle formulations offer advantages in this regard, and various nanoparticle systems for siRNA delivery have been developed for various applications. However, considering the 3R principle as well as experimental limitations, it is not possible to test all different nanoparticle systems potentially useful for lung application directly in vivo. ALI cultures thus represent models for faithfully recapitulating several of the above requirements, while offering a more straightforward test system.

For example, cationic receptor-targeted nanocomplex translocation through mucus and transfection efficiencies were studied in primary CF epithelial cells grown under ALI conditions, for knockdown of the epithelial sodium channel (ENaC). Concomitantly, nanocomplex activity was also observed in vivo after oropharyngeal instillation [65]. This indicates that ALI culture results may predict in vivo activities. In an ALI culture on Calu-3 cells, profound knockdown was observed upon transfection with a GAPDH-specific siRNA, using a VIPER (virus-inspired polymer for endosomal release) block copolymer system [66]. This was already seen at 24 h, indicating that knockdown induced by nanoparticle-mediated siRNA delivery may be a rapid event even in ALI culture, at least on the mRNA level and for certain target genes. 

While our results demonstrate that 2D nanoparticle efficacies cannot readily predict efficacies in ALI culture, the previously described strong improvement in knockdown efficacy and biocompatibility after tyrosine grafting [40,42] was also seen in the ALI system, at least in the case of some polymers. On the other hand, major differences were found in the case of LP10Y/siRNA polyplexes prepared in HN buffer, exhibiting high transfection efficacy in 2D cell culture, but not in the physiologically more relevant ALI model. This is one example demonstrating the benefit of different test systems to evaluate nanoparticles prior to in vivo testing. Likewise, due to its lack of activity in the ALI despite high knockdown efficacy in 2D cell culture, P10F was not considered as similarly promising for pulmonary application as some tyrosine-grafted PEIs.

Moreover, knockdown efficacies were found to be dependent on complexation conditions, nanoparticle hydrodynamic diameter and ζ-potential. Notably, some of our nanoparticles were found to be quite large, while still exerting transfection efficacy. Previously, we and others have already quite extensively looked into the phenomenon of large(r) nanoparticle uptake and transfection efficacy. Indeed, it was found that PEI nanoparticle transfection efficacies benefit from larger size (see e.g., [67]). Significant increases in gene expression activity after transfection with larger particles were found under standard in vitro culture conditions. The uptake mechanisms of these larger PEI-based nanoparticles include macropinocytosis [68] as well as classical clathrin- or caveolin-dependent endocytosis [40,69], as determined by the co-treatment of cells with different inhibitors influencing cellular uptake and/or endosomal escape. Again, these particles also showed high biocompatibility and no LDH release over basal levels. While in some of these studies DNA-containing PEI complexes were studied, similar results were also found in the case of siRNA-containing particles (approximately 300 nm diameter) [40,42]. Notably, however, these experiments were conducted in classical 2D cell culture, where enhanced transfection efficacies may also benefit from increased/more rapid nanoparticle sedimentation onto cell growth adjacently on the bottom of the culture dish. This highlights again the need for other models (like the one described here) to analyze transfection efficacies more comprehensively.

The hydrodynamic diameter and ζ-potential, however, do not seem to be the only critical variables, with additional effects like the formation of a nanoparticle corona upon exposure to the environment likely contributing as well. It has been well established that the interaction of the particle with the medium leads to the formation of a protein corona which can alter the particle characteristics and behavior [15,16,70]. Thus, the ζ-potential of nanoparticles initially measured in buffer may be even less important, bearing in mind that all particles show negative potentials after incubation with culture medium. Since the cells will interact with these “medium-coated” particles rather than with the free nanoparticle, we assume that the “buffer-only” ζ-potentials may play an only minor role. Beyond proteins, this corona may also contain other biomolecules like lipids or components from the mucus or surfactant. Indeed, it was shown previously that coating of nanoparticles based on 25 kDa PEI with Alveofact enhanced their knockdown efficacy in 16HBE ALI cultures on GAPDH mRNA level [39]. Thus, ALI cultures, rather than classical 2D cell culture, are suitable models for further characterizing the influence of a given nanoparticle corona, for example by directly exposing nanoparticles to biomolecules prior to ALI culture transfection and the subsequent analysis of their biological activity and cytotoxicity. 

Beyond corona formation, nanoparticle stability and penetration is a major issue as well. Indeed, a main problem in the context of pulmonary application appears to be the interaction of the nanoparticle with the mucus lining the upper airways or the pulmonary surfactant, predominantly localized in the lower airways. Mucus represents a viscoelastic gel containing a mixture of glycoproteins, non-mucin proteins, salts, lipids, DNA, cells and cellular debris in water. Several of these components can interact with a given nanoparticle and potentially impair its stability. This may be particularly true for pathologic mucus compositions represented, for example, by cystic fibrosis artificial mucus [71,72,73]. In contrast, surfactant in the alveolar region, with the main ingredient being DPPC [74,75], may be considered as less problematic, because particle integrity may not be as substantially affected. Indeed, in our studies, comparable nanoparticle activities upon surfactant pre-incubation were seen for (L)P10Y/siRNA polyplexes or DPPC-(L)P10Y/siRNA lipopolyplexes in classical 2D cell culture. In contrast, however, differences in activity seen in the ALI system do indicate differences in particle characteristics, which influence the biological activity in the more complex system. Between the efficient nanoparticles in the ALI culture system, we detected some mismatches between the cellular siRNA uptake and the resulting knockdown efficacies. This indicates that other factors like the intracellular release of the nanoparticles, and/or intracellular nanoparticle decomposition with subsequent siRNA release may well contribute to overall knockdown efficacy. Further experiments will be necessary to elucidate these differences in particle behavior and biological properties.

The use of an ALI model is a comparatively simple way to more closely mimic the local application of a drug to the lung compared to standard cell culture. This relies on the formation of a relatively tight cell layer with substantially more cell–cell interactions than in 2D cell culture, where more or less single cells are treated. Furthermore, the cell density is more representative of the situation in the lung, and cytotoxic effects are often inversely correlated with cell density in cell culture. The ALI system is easier to establish than, for example, organoid structures or lung tissue slices. In further experiments, the testing of promising candidates from ALI experiments should be further pursued in more complex systems, including cell co-culture systems (ALI) relying on different cell types, organoid models or lung slices. While some systemic effects like a hepatic first pass effect or interactions with endothelial layers may not be a major concern in the context of local delivery to the lung, effects on the lung epithelium or immune cells populating the alveolar space will have to be considered. Notably, these effects on different cell populations of the lung could also be analyzed using co-culture models, e.g., comprising epithelial cells and immune cells [25,26] or epithelial cells and fibroblasts [27]. Commercially available multi-cell-type ALI models already provide relatively standardized platforms of multicellular test systems for different parts of the respiratory tract [17].

Taken together, the 3D ALI cell model provides the basis for a more realistic assessment of biological nanoparticle effects, as compared to 2D cell culture. This allows for the definition of optimal nanoparticles in terms of their composition, as well as their preparation. Furthermore, the development of an inhalable particle formulation is a crucial step for local in vivo application into the lung. Notably, the data already show that particles described in this paper remain stable and active after aerosol formation using an Aeroneb X-Pro nebulizer [76], and unpublished data for after spray-drying [54].

## Figures and Tables

**Table 1:** Hydrodynamic diameters and ζ-potentials of complexes, prepared in standard cell culture complexation buffer (HN) or in 10% trehalose containing 20 mM Hepes (Tre).

Complex	Complexation Buffer	Hydrodynamic Diameter (nm) ± SD	ζ-Potential (mV) ± SD
P10Y/siLuc2	HN	472 ± 183	−10 ± 3
	Tre	345 ± 133	−10 ± 1
DPPC-P10Y/siLuc2	HN	129 ± 24	−1 ± 1
	Tre	255 ± 92	−0.4 ± 0.4
LP10Y/siLuc2	HN	239 ± 101	29.6 ± 0.3
	Tre	68 ± 1	25 ± 2
LP10Y/siLuc2 (1 µg/10 µL)	Tre	127 ± 19	26.4 ± 0.9
DPPC-LP10Y/siLuc2	HN	138 ± 3	7 ± 1
	Tre	138 ± 12	10 ± 3
DPPC-LP10Y/siLuc2 (1 µg/10 µL)	Tre	144 ± 10	15 ± 5
P10/siLuc2	HN	237 ± 43	33 ± 1
	Tre	72 ± 8	19 ± 2
P10F/siLuc2	HN	188 ± 97	9 ± 6
	Tre	291 ± 110	20 ± 1

## Data Availability

Dataset available on request from the authors.

## Supplementary Materials

The following supporting information can be downloaded at: https://www.mdpi.com/article/10.3390/pharmaceutics17030339/s1, Figure S1: Fluoroalkyl modification of 10 kDa branched PEI (P10). (A) Synthesis scheme; (B) attempted and obtained degree of fluoroalkyl modification, as determined from the ^1^H NMR spectra of the P10F50 (C), P10F25 (D) and P10F5 (E); Figure S2: Knockdown efficacies of complexes based on the different P10F derivatives and prepared at different mass ratios, as determined in H441-Luc cells. Data are reported as mean ± SD; Figure S3: Panel of nanoparticles tested for EGFP knockdown efficiencies in A549-EGFP/Luc ALI cell cultures. For promising candidates, a dose reduction to 1 μg was included as well. Data are reported as mean ± SEM. Significance indicated by * = *p* < 0.03; ** = *p* < 0.01; *** = *p* < 0.001 between negative control and specific siRNA transfection; Figure S4: Dose-dependent cellular uptake of a fluorescence-labeled siRNA in A549-eGFP/Luc ALI culture 24 h after transfection using LP10Y (left) or P10Y (right), shown as (A) increase in mean fluorescence intensity and (B) in the corresponding original flow cytometry histograms. (C) EGFP knockdown efficacies after 96 h, determined by flow cytometry. (D) Impact of particle-medium-interaction on the hydrodynamic diameter of (lipo)polyplexes. Data are reported as mean ± SEM. Significance indicated by * = *p* < 0.03; ** = *p* < 0.01; *** = *p* < 0.001 between negative control (siCtrl) and specific siRNA transfection; Table S1: Mass ratios and corresponding N/P ratios employed for complexation; Table S2: siRNAs used in this study.

## Abbreviations

The following abbreviations are used in this manuscript:

ALI	Air–liquid interface
DPPC	Dipalmitoylphosphatidylcholine
EGFP	enhanced green fluorescent protein
LDH	Lactate dehydrogenase
LPP	Lipopolyplex
Luc	Luciferase
LP10	Linear 10 kDa PEI
LP10Y	Tyrosine-modified linear 10 kDa PEI
PEI	Polyethylenimine
pos-ctrl	positive control
P10	10 kDa branched PEI
P10F	Fluoroalkyl-modified 10 kDa PEI
P10Y	Tyrosine-modified 10 kDa PEI
RFU	Relative fluorescence units
RLU	Relative light units
siCtrl	Negative control siRNA
siRNA	Small interfering RNA
TEM	Transmission electron microscopy

## References

[1] Corkery K. (2000). Inhalable drugs for systemic therapy. Respir. Care.

[2] Paranjpe M., Müller-Goymann C.C. (2014). Nanoparticle-mediated pulmonary drug delivery: A review. Int. J. Mol. Sci..

[3] Patton J.S. (1996). Mechanisms of macromolecule absorption by the lungs. Adv. Drug Deliv. Rev..

[4] Labiris N.R., Dolovich M.B. (2003). Pulmonary drug delivery. Part I: Physiological factors affecting therapeutic effectiveness of aerosolized medications. Br. J. Clin. Pharmacol..

[5] Upton R.N., Doolette D.J. (1999). Kinetic aspects of drug disposition in the lungs. Clin. Exp. Pharmacol. Physiol..

[6] Heijerman H., Westerman E., Conway S., Touw D., Döring G. (2009). Inhaled medication and inhalation devices for lung disease in patients with cystic fibrosis: A European consensus. J. Cyst. Fibros..

[7] Alsaeedi A., Sin D.D., McAlister F.A. (2002). The effects of inhaled corticosteroids in chronic obstructive pulmonary disease: A systematic review of randomized placebo-controlled trials. Am. J. Med..

[8] Kuzmov A., Minko T. (2015). Nanotechnology approaches for inhalation treatment of lung diseases. J. Control. Release.

[9] Shepard K.B., Vodak D.T., Kuehl P.J., Revelli D., Zhou Y., Pluntze A.M., Adam M.S., Oddo J.C., Switala L., Cape J.L. (2021). Local Treatment of Non-small Cell Lung Cancer with a Spray-Dried Bevacizumab Formulation. AAPS PharmSciTech.

[10] Aufderheide M., Förster C., Beschay M., Branscheid D., Emura M. (2016). A new computer-controlled air-liquid interface cultivation system for the generation of differentiated cell cultures of the airway epithelium. Exp. Toxicol. Pathol..

[11] Stewart C.E., Torr E.E., Mohd Jamili N.H., Bosquillon C., Sayers I. (2012). Evaluation of differentiated human bronchial epithelial cell culture systems for asthma research. J. Allergy.

[12] Braakhuis H.M., Kloet S.K., Kezic S., Kuper F., Park M.V.D.Z., Bellmann S., van der Zande M., Le Gac S., Krystek P., Peters R.J.B. (2015). Progress and future of in vitro models to study translocation of nanoparticles. Arch. Toxicol..

[13] Zscheppang K., Berg J., Hedtrich S., Verheyen L., Wagner D.E., Suttorp N., Hippenstiel S., Hocke A.C. (2018). Human Pulmonary 3D Models For Translational Research. Biotechnol. J..

[14] Leach T., Gandhi U., Reeves K.D., Stumpf K., Okuda K., Marini F.C., Walker S.J., Boucher R., Chan J., Cox L.A. (2023). Development of a novel air-liquid interface airway tissue equivalent model for in vitro respiratory modeling studies. Sci. Rep..

[15] Ahmad S., Ahmad A., Neeves K.B., Hendry-Hofer T., Loader J.E., White C.W., Veress L. (2014). In vitro cell culture model for toxic inhaled chemical testing. J. Vis. Exp..

[16] Limbach L.K., Li Y., Grass R.N., Brunner T.J., Hintermann M.A., Muller M., Gunther D., Stark W.J. (2005). Oxide nanoparticle uptake in human lung fibroblasts: Effects of particle size, agglomeration, and diffusion at low concentrations. Environ. Sci. Technol..

[17] Baldassi D., Gabold B., Merkel O. (2021). Air-liquid interface cultures of the healthy and diseased human respiratory tract: Promises, challenges and future directions. Adv. Nanobiomed. Res..

[18] Reichl S., Becker K. (2012). Cultivation of RPMI 2650 cells as an in-vitro model for human transmucosal nasal drug absorption studies: Optimization of selected culture conditions. J. Pharm. Pharmacol..

[19] Kreft M.E., Jerman U.D., Lasič E., Lanišnik Rižner T., Hevir-Kene N., Peternel L., Kristan K. (2015). The characterization of the human nasal epithelial cell line RPMI 2650 under different culture conditions and their optimization for an appropriate in vitro nasal model. Pharm. Res..

[20] Barlang L.-A., Weinbender K., Merkel O.M., Popp A. (2024). Characterization of critical parameters using an air-liquid interface model with RPMI 2650 cells for permeability studies of small molecules. Drug Deliv. Transl. Res..

[21] Sanchez-Guzman D., Boland S., Brookes O., Mc Cord C., Lai Kuen R., Sirri V., Baeza Squiban A., Devineau S. (2021). Long-term evolution of the epithelial cell secretome in preclinical 3D models of the human bronchial epithelium. Sci. Rep..

[22] Callaghan P.J., Ferrick B., Rybakovsky E., Thomas S., Mullin J.M. (2020). Epithelial barrier function properties of the 16HBE14o-human bronchial epithelial cell culture model. Biosci. Rep..

[23] Ren H., Birch N.P., Suresh V. (2016). An Optimised Human Cell Culture Model for Alveolar Epithelial Transport. PLoS ONE.

[24] Blank F., Rothen-Rutishauser B.M., Schurch S., Gehr P. (2006). An optimized in vitro model of the respiratory tract wall to study particle cell interactions. J. Aerosol Med..

[25] Braakhuis H.M., Gremmer E.R., Bannuscher A., Drasler B., Keshavan S., Rothen-Rutishauser B., Birk B., Verlohner A., Landsiedel R., Meldrum K. (2023). Transferability and reproducibility of exposed air-liquid interface co-culture lung models. NanoImpact.

[26] He R.-W., Braakhuis H.M., Vandebriel R.J., Staal Y.C.M., Gremmer E.R., Fokkens P.H.B., Kemp C., Vermeulen J., Westerink R.H.S., Cassee F.R. (2021). Optimization of an air-liquid interface in vitro cell co-culture model to estimate the hazard of aerosol exposures. J. Aerosol Sci..

[27] Barron S.L., Wyatt O., O’Connor A., Mansfield D., Suzanne Cohen E., Witkos T.M., Strickson S., Owens R.M. (2024). Modelling bronchial epithelial-fibroblast cross-talk in idiopathic pulmonary fibrosis (IPF) using a human-derived in vitro air liquid interface (ALI) culture. Sci. Rep..

[28] Viana F., O’Kane C.M., Schroeder G.N. (2022). Precision-cut lung slices: A powerful ex vivo model to investigate respiratory infectious diseases. Mol. Microbiol..

[29] Barosova H., Meldrum K., Karakocak B.B., Balog S., Doak S.H., Petri-Fink A., Clift M.J.D., Rothen-Rutishauser B. (2021). Inter-laboratory variability of A549 epithelial cells grown under submerged and air-liquid interface conditions. Toxicol. Vitr..

[30] Tanabe I., Ishimori K., Ishikawa S. (2024). Development of an in vitro human alveolar epithelial air-liquid interface model using a small molecule inhibitor cocktail. BMC Mol. Cell Biol..

[31] Caraballo J.C., Yshii C., Westphal W., Moninger T., Comellas A.P. (2011). Ambient particulate matter affects occludin distribution and increases alveolar transepithelial electrical conductance. Respirology.

[32] Gerovac B.J., Valencia M., Baumlin N., Salathe M., Conner G.E., Fregien N.L. (2014). Submersion and hypoxia inhibit ciliated cell differentiation in a notch-dependent manner. Am. J. Respir. Cell Mol. Biol..

[33] Öhlinger K., Kolesnik T., Meindl C., Gallé B., Absenger-Novak M., Kolb-Lenz D., Fröhlich E. (2019). Air-liquid interface culture changes surface properties of A549 cells. Toxicol. Vitr..

[34] Endter S., Francombe D., Ehrhardt C., Gumbleton M. (2009). RT-PCR analysis of ABC, SLC and SLCO drug transporters in human lung epithelial cell models. J. Pharm. Pharmacol..

[35] Bañuls L., Pellicer D., Castillo S., Navarro-García M.M., Magallón M., González C., Dasí F. (2020). Gene Therapy in Rare Respiratory Diseases: What Have We Learned So Far?. J. Clin. Med..

[36] Setten R.L., Rossi J.J., Han S.-P. (2019). The current state and future directions of RNAi-based therapeutics. Nat. Rev. Drug Discov..

[37] Lewis M.M., Soto M.R., Maier E.Y., Wulfe S.D., Bakheet S., Obregon H., Ghosh D. (2023). Optimization of ionizable lipids for aerosolizable mRNA lipid nanoparticles. Bioeng. Transl. Med..

[38] Conte G., Costabile G., Baldassi D., Rondelli V., Bassi R., Colombo D., Linardos G., Fiscarelli E.V., Sorrentino R., Miro A. (2022). Hybrid Lipid/Polymer Nanoparticles to Tackle the Cystic Fibrosis Mucus Barrier in siRNA Delivery to the Lungs: Does PEGylation Make the Difference?. ACS Appl. Mater. Interfaces.

[39] Baldassi D., Ngo T.M.H., Merkel O.M. (2024). Optimization of Lung Surfactant Coating of siRNA Polyplexes for Pulmonary Delivery. Pharm. Res..

[40] Ewe A., Przybylski S., Burkhardt J., Janke A., Appelhans D., Aigner A. (2016). A novel tyrosine-modified low molecular weight polyethylenimine (P10Y) for efficient siRNA delivery in vitro and in vivo. J. Control. Release.

[41] Ewe A., Noske S., Karimov M., Aigner A. (2019). Polymeric Nanoparticles Based on Tyrosine-Modified, Low Molecular Weight Polyethylenimines for siRNA Delivery. Pharmaceutics.

[42] Karimov M., Schulz M., Kahl T., Noske S., Kubczak M., Gockel I., Thieme R., Büch T., Reinert A., Ionov M. (2021). Tyrosine-modified linear PEIs for highly efficacious and biocompatible siRNA delivery in vitro and in vivo. Nanomedicine.

[43] Tian X.-L., Chen P., Hu Y., Zhang L., Yu X.-Q., Zhang J. (2024). Enhanced gene transfection ability of sulfonylated low-molecular-weight PEI and its application in anti-tumor treatment. J. Mater. Chem. B.

[44] Zhou X., Cao Y., Li R., Di X., Wang Y., Wang K. (2024). PEI, a new transfection method, augments the inhibitory effect of RBM5 on prostate cancer. Biochem. Biophys. Res. Commun..

[45] Kubczak M., Michlewska S., Karimov M., Ewe A., Noske S., Aigner A., Bryszewska M., Ionov M. (2022). Unmodified and tyrosine-modified polyethylenimines as potential carriers for siRNA: Biophysical characterization and toxicity. Int. J. Pharm..

[46] Kwolek U., Jamróz D., Janiczek M., Nowakowska M., Wydro P., Kepczynski M. (2016). Interactions of Polyethylenimines with Zwitterionic and Anionic Lipid Membranes. Langmuir.

[47] Casper J., Schenk S.H., Parhizkar E., Detampel P., Dehshahri A., Huwyler J. (2023). Polyethylenimine (PEI) in gene therapy: Current status and clinical applications. J. Control. Release.

[48] Noske S., Karimov M., Aigner A., Ewe A. (2020). Tyrosine-Modification of Polypropylenimine (PPI) and Polyethylenimine (PEI) Strongly Improves Efficacy of siRNA-Mediated Gene Knockdown. Nanomaterials.

[49] Keshavarz V., Kazemi M., Khalvati B., Zare F., Dehshahri A., Sadeghpour H. (2024). Surface decoration of low molecular weight polyethylenimine (LMW PEI) by phthalated dextrin for improved delivery of interleukin-12 plasmid. Biotechnol. Prog..

[50] Borchardt H., Ewe A., Morawski M., Weirauch U., Aigner A. (2021). miR24-3p activity after delivery into pancreatic carcinoma cell lines exerts profound tumor-inhibitory effects through distinct pathways of apoptosis and autophagy induction. Cancer Lett..

[51] Ewe A., Höbel S., Heine C., Merz L., Kallendrusch S., Bechmann I., Merz F., Franke H., Aigner A. (2017). Optimized polyethylenimine (PEI)-based nanoparticles for siRNA delivery, analyzed in vitro and in an ex vivo tumor tissue slice culture model. Drug Deliv. Transl. Res..

[52] Noske S., Karimov M., Hansen M., Zatula N., Ewe A., Aigner A. (2022). Non-viral siRNA transfection of primary mesenchymal stromal cells (MSCs): Assessment of tyrosine-modified PEI and PPI efficacy and biocompatibility. Int. J. Pharm..

[53] Merz L., Höbel S., Kallendrusch S., Ewe A., Bechmann I., Franke H., Merz F., Aigner A. (2017). Tumor tissue slice cultures as a platform for analyzing tissue-penetration and biological activities of nanoparticles. Eur. J. Pharm. Biopharm..

[54] Noske S., Karimov M., Krüger M., Lilli B., Ewe A., Aigner A. (2024). Spray-drying of PEI-/PPI-based nanoparticles for DNA or siRNA delivery. Eur. J. Pharm. Biopharm..

[55] Schäfer J., Höbel S., Bakowsky U., Aigner A. (2010). Liposome-polyethylenimine complexes for enhanced DNA and siRNA delivery. Biomaterials.

[56] Ewe A., Schaper A., Barnert S., Schubert R., Temme A., Bakowsky U., Aigner A. (2014). Storage stability of optimal liposome-polyethylenimine complexes (lipopolyplexes) for DNA or siRNA delivery. Acta Biomater..

[57] Johnson M.E., Shon J., Guan B.M., Patterson J.P., Oldenhuis N.J., Eldredge A.C., Gianneschi N.C., Guan Z. (2016). Fluorocarbon Modified Low-Molecular-Weight Polyethylenimine for siRNA Delivery. Bioconjug. Chem..

[58] Jin Y., Yu W., Zhang W., Wang C., Liu Y., Yuan W.-E., Feng Y. (2023). A novel fluorinated polyethyleneimine with microRNA-942-5p-sponges polyplex gene delivery system for non-small-cell lung cancer therapy. J. Colloid Interface Sci..

[59] Rosas-Taraco A.G., Higgins D.M., Sánchez-Campillo J., Lee E.J., Orme I.M., González-Juarrero M. (2011). Local pulmonary immunotherapy with siRNA targeting TGFβ1 enhances antimicrobial capacity in Mycobacterium tuberculosis infected mice. Tuberculosis.

[60] D’Alessandro-Gabazza C.N., Kobayashi T., Boveda-Ruiz D., Takagi T., Toda M., Gil-Bernabe P., Miyake Y., Yasukawa A., Matsuda Y., Suzuki N. (2012). Development and preclinical efficacy of novel transforming growth factor-β1 short interfering RNAs for pulmonary fibrosis. Am. J. Respir. Cell Mol. Biol..

[61] Goh F.Y., Cook K.L.T.P., Upton N., Tao L., Lah L.C., Leung B.P., Wong W.S.F. (2013). Receptor-interacting protein 2 gene silencing attenuates allergic airway inflammation. J. Immunol..

[62] Miwata K., Okamoto H., Nakashima T., Ihara D., Horimasu Y., Masuda T., Miyamoto S., Iwamoto H., Fujitaka K., Hamada H. (2018). Intratracheal Administration of siRNA Dry Powder Targeting Vascular Endothelial Growth Factor Inhibits Lung Tumor Growth in Mice. Mol. Ther. Nucleic Acids.

[63] Ding L., Tang S., Wyatt T.A., Knoell D.L., Oupický D. (2021). Pulmonary siRNA delivery for lung disease: Review of recent progress and challenges. J. Control. Release.

[64] Zoulikha M., Xiao Q., Boafo G.F., Sallam M.A., Chen Z., He W. (2022). Pulmonary delivery of siRNA against acute lung injury/acute respiratory distress syndrome. Acta Pharm. Sin. B.

[65] Tagalakis A.D., Munye M.M., Ivanova R., Chen H., Smith C.M., Aldossary A.M., Rosa L.Z., Moulding D., Barnes J.L., Kafetzis K.N. (2018). Effective silencing of ENaC by siRNA delivered with epithelial-targeted nanocomplexes in human cystic fibrosis cells and in mouse lung. Thorax.

[66] Baldassi D., Ambike S., Feuerherd M., Cheng C.-C., Peeler D.J., Feldmann D.P., Porras-Gonzalez D.L., Wei X., Keller L.-A., Kneidinger N. (2022). Inhibition of SARS-CoV-2 replication in the lung with siRNA/VIPER polyplexes. J. Control. Release.

[67] Pezzoli D., Giupponi E., Mantovani D., Candiani G. (2017). Size matters for in vitro gene delivery: Investigating the relationships among complexation protocol, transfection medium, size and sedimentation. Sci. Rep..

[68] Zhang W., Kang X., Yuan B., Wang H., Zhang T., Shi M., Zheng Z., Zhang Y., Peng C., Fan X. (2019). Nano-Structural Effects on Gene Transfection: Large, Botryoid-Shaped Nanoparticles Enhance DNA Delivery via Macropinocytosis and Effective Dissociation. Theranostics.

[69] Karimov M., Appelhans D., Ewe A., Aigner A. (2021). The combined disulfide cross-linking and tyrosine-modification of very low molecular weight linear PEI synergistically enhances transfection efficacies and improves biocompatibility. Eur. J. Pharm. Biopharm..

[70] Zhu D., Yan H., Zhou Z., Tang J., Liu X., Hartmann R., Parak W.J., Feliu N., Shen Y. (2018). Detailed investigation on how the protein corona modulates the physicochemical properties and gene delivery of polyethylenimine (PEI) polyplexes. Biomater. Sci..

[71] Sonntag T., Rapp M., Didier P., Lebeau L., Pons F., Casset A. (2022). Mucus-producing epithelial models for investigating the activity of gene delivery systems in the lung. Int. J. Pharm..

[72] Yang Y., Tsifansky M.D., Wu C.-J., Yang H.I., Schmidt G., Yeo Y. (2010). Inhalable antibiotic delivery using a dry powder co-delivering recombinant deoxyribonuclease and ciprofloxacin for treatment of cystic fibrosis. Pharm. Res..

[73] Porsio B., Craparo E.F., Mauro N., Giammona G., Cavallaro G. (2018). Mucus and Cell-Penetrating Nanoparticles Embedded in Nano-into-Micro Formulations for Pulmonary Delivery of Ivacaftor in Patients with Cystic Fibrosis. ACS Appl. Mater. Interfaces.

[74] Possmayer F., Zuo Y.Y., Veldhuizen R.A.W., Petersen N.O. (2023). Pulmonary Surfactant: A Mighty Thin Film. Chem. Rev..

[75] Bernhard W. (2016). Lung surfactant: Function and composition in the context of development and respiratory physiology. Ann. Anat..

[76] Ewe A., Aigner A. (2014). Nebulization of liposome–polyethylenimine complexes (lipopolyplexes) for DNA or siRNA delivery: Physicochemical properties and biological activity. Euro. J. Lipid Sci. Tech..

