# Supplementary material for: Analysis of Polymer/siRNA Nanoparticle Efficacy and Biocompatibility in 3D Air–Liquid Interface Culture Compared to 2D Cell Culture
Source: Pharmaceutics. 2025 Mar 6;17(3):339. doi: 10.3390/pharmaceutics17030339 (PMC11946471; doi:10.3390/pharmaceutics17030339)
Supplement: Supplementary file 1 [file pharmaceutics-17-00339-s001.zip › Figure S1.pdf]

A

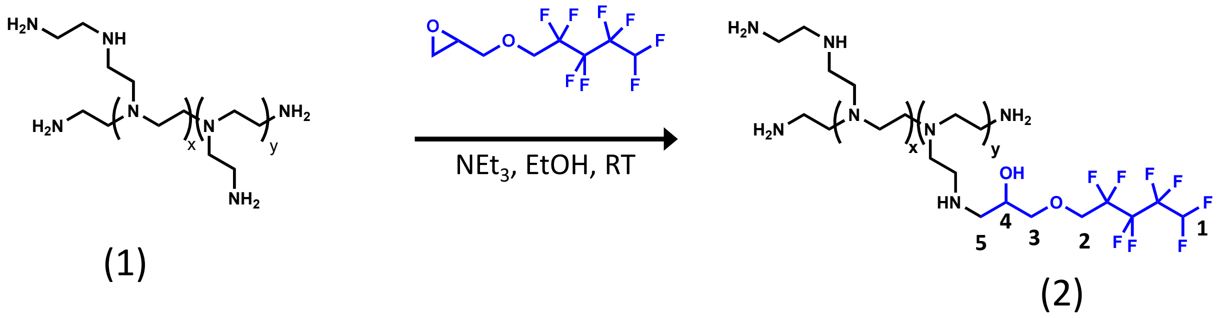

B

P10F derivative	% of fluoroalkyl modification	
	attempted	obtained
$P10F_{50}$	50%	64.5%
$P10F_{25}$	25%	27.5%
$P10F_{5}$	5%	5.1%

C

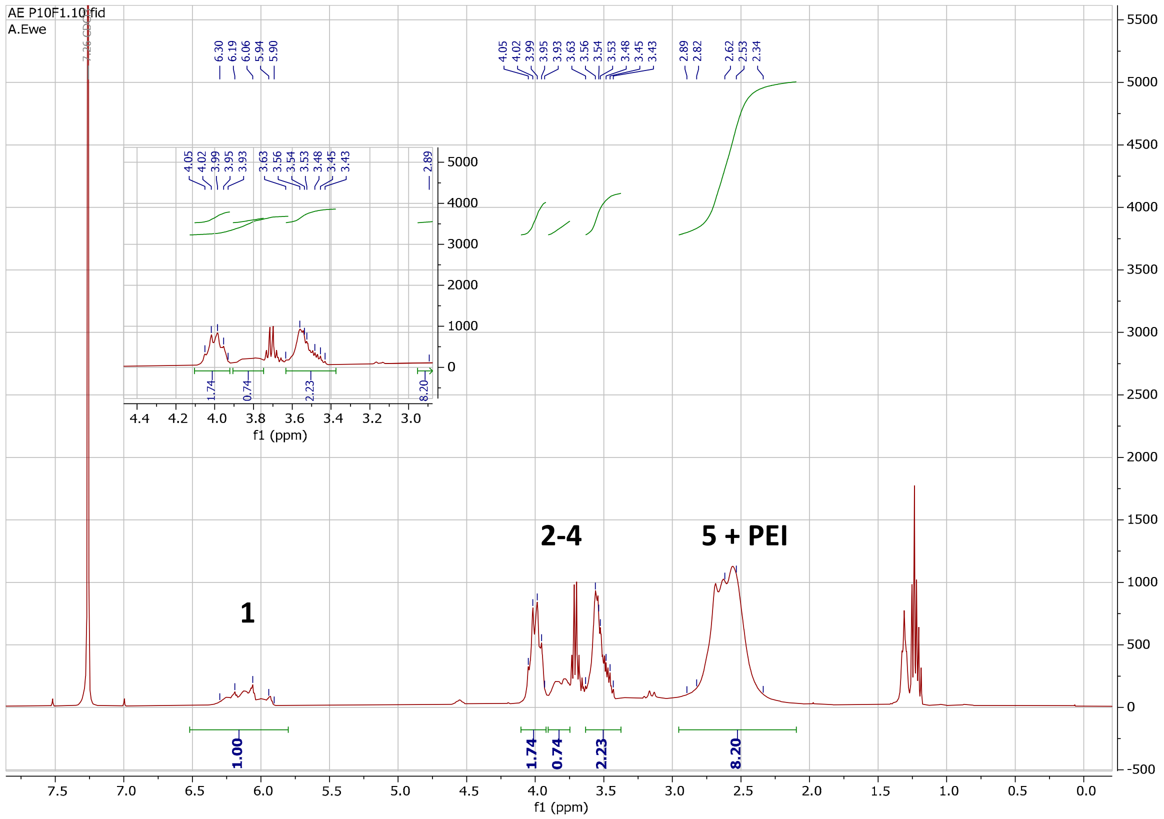

Figure S1

D

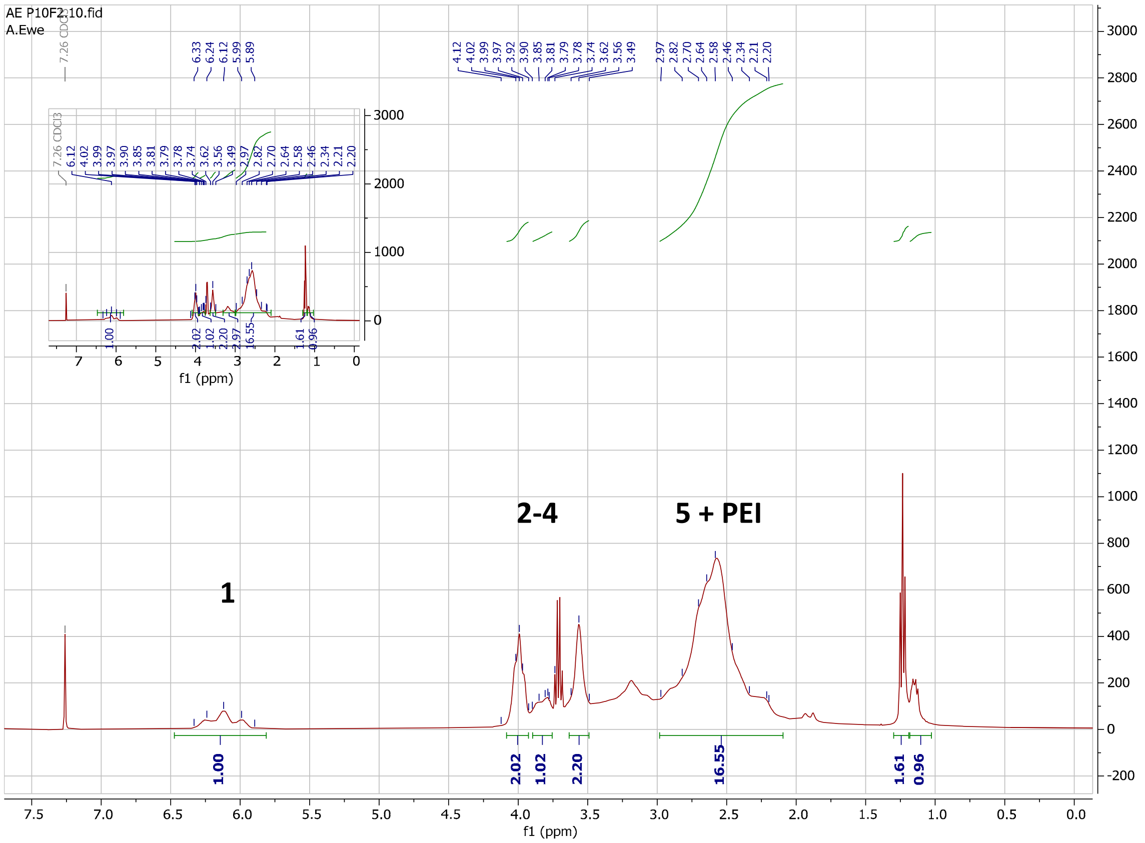
AE P10F2.10.fid
A.Ewe
1
2-4
5 + PEI
1.00
2.02
1.02
2.20
16.55
1.61
0.96
f1 (ppm)

E

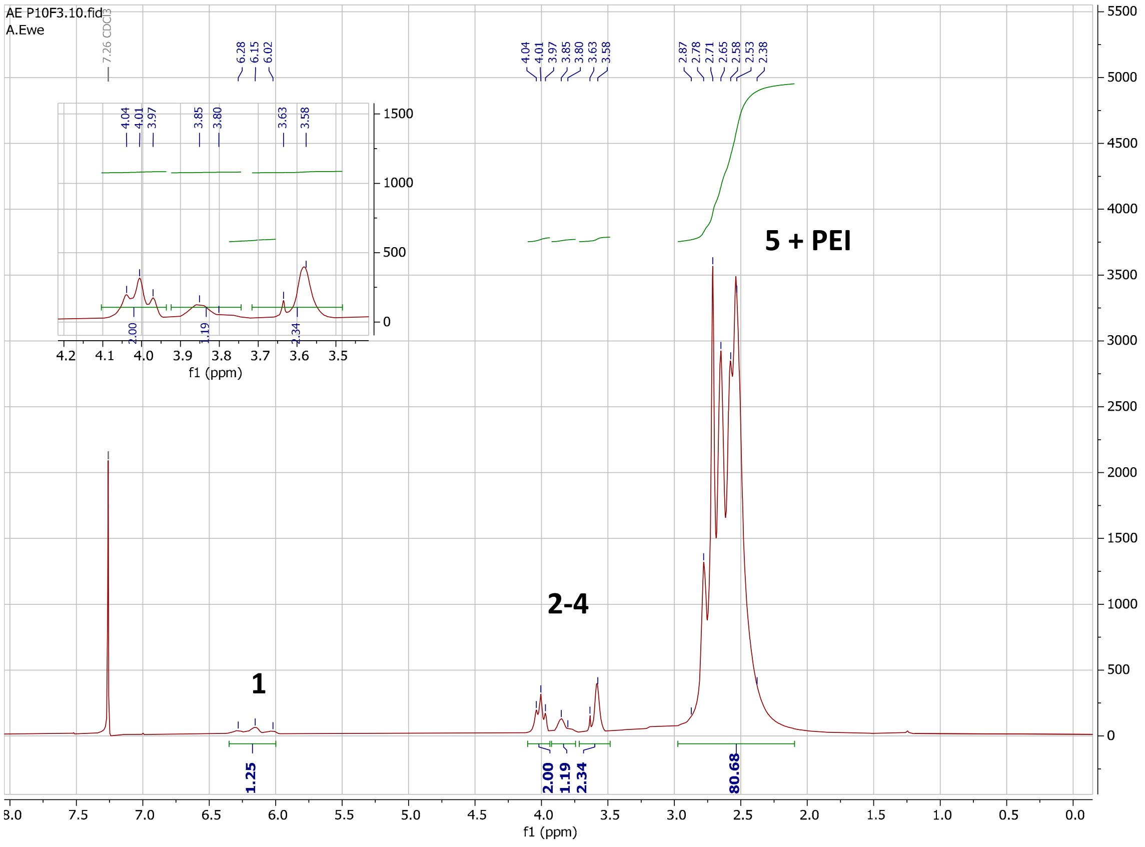
AE P10F3.10.fid
A.Ewe
1
2-4
5 + PEI
1.25
2.00
1.19
2.34
80.68
f1 (ppm)

Figure S1
