# Supplementary material for: Analysis of Polymer/siRNA Nanoparticle Efficacy and Biocompatibility in 3D Air–Liquid Interface Culture Compared to 2D Cell Culture
Source: Pharmaceutics. 2025 Mar 6;17(3):339. doi: 10.3390/pharmaceutics17030339 (PMC11946471; doi:10.3390/pharmaceutics17030339)
Supplement: Supplementary file 1 [file pharmaceutics-17-00339-s001.zip › Table S1.pdf]

Table S1

Mass ratios and corresponding N/P ratios employed for complexation.

Polymer	Mass ratio used in this study	N/P ratio
$P10F_{50}$	2.5	3.0
$P10F_{25}$	2.5	6.8
$P10F_{5}$	2.5	14.3
P10Y	2.5	8.5
LP10Y	2.5	8.5
P10	7.5	57.6
