# Supplementary material for: Analysis of Polymer/siRNA Nanoparticle Efficacy and Biocompatibility in 3D Air–Liquid Interface Culture Compared to 2D Cell Culture
Source: Pharmaceutics. 2025 Mar 6;17(3):339. doi: 10.3390/pharmaceutics17030339 (PMC11946471; doi:10.3390/pharmaceutics17030339)
Supplement: Supplementary file 1 [file pharmaceutics-17-00339-s001.zip › Table S2.pdf]

Table S2

siRNAs used in this study

siRNA	sequence 5‘ - 3‘		Vendor
siLuc2	sense	CGUACGCGGAAUACUUCGA dTdT	Dharmacon, Lafayette, CO, USA
	antisense	UCGAAGUAUUCCGCGUACG dTdT	
siGAPDH	sense	CCUCAACUACAUGGUUUAC dTdT	Eurogentec, Seraing, Belgium
	antisense	GUAAACCAUGUAGUUGAGG dTdT	
siLuc3	sense	CUUACGCUGAGUACUUCGA dTdT	Eurogentec, Seraing, Belgium
	antisense	UCGAAGUACUCAGCGUAAG dTdT	
siEGFP	sense	GCAGCACGACUUCUUCAAG dTdT	Eurogentec, Seraing, Belgium
	antisense	CUUGAAGAAGUCGUGCUGC dTdT	
HiLyte647-siEGFP	sense	HiLyte 647-GCAGCACGACUUCUUCAAG dTdT	Eurogentec, Seraing, Belgium
	antisense	CUUGAAGAAGUCGUGCUGC dTdT	
