# Supplementary figures and images for: Analysis of Polymer/siRNA Nanoparticle Efficacy and Biocompatibility in 3D Air–Liquid Interface Culture Compared to 2D Cell Culture
Source: Pharmaceutics. 2025 Mar 6;17(3):339. doi: 10.3390/pharmaceutics17030339 (PMC11946471; doi:10.3390/pharmaceutics17030339)

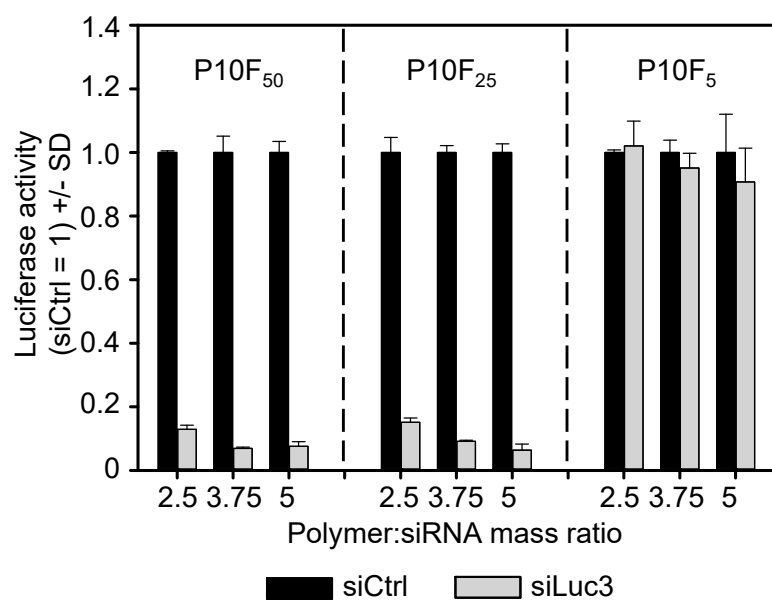

Figure S2

Supplement: Supplementary file 1 [file pharmaceutics-17-00339-s001.zip › Figure S2.pdf]

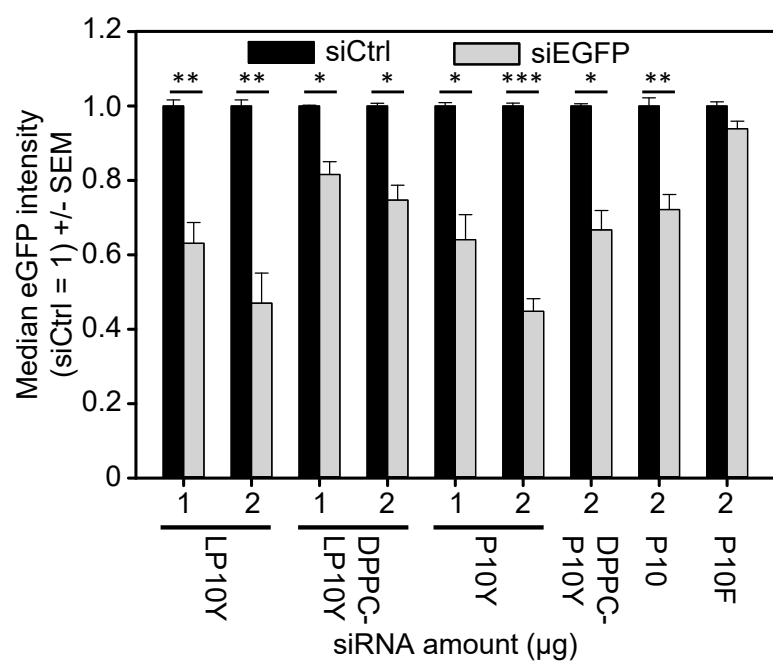

Figure S3

Supplement: Supplementary file 1 [file pharmaceutics-17-00339-s001.zip › Figure S3.pdf]

A

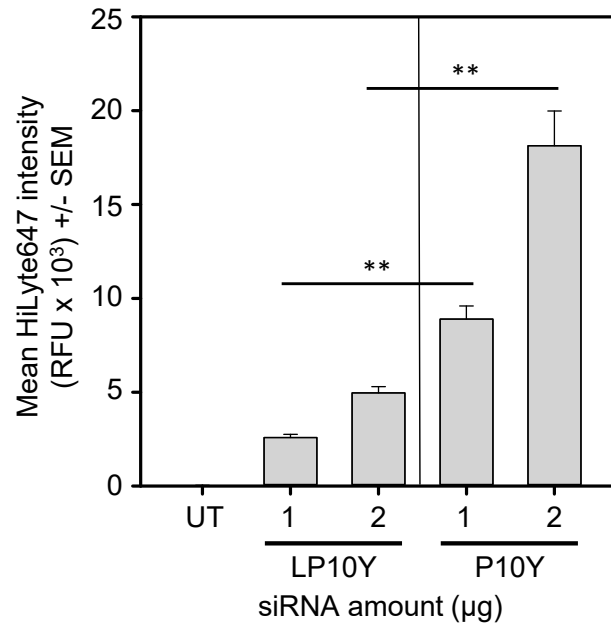

B

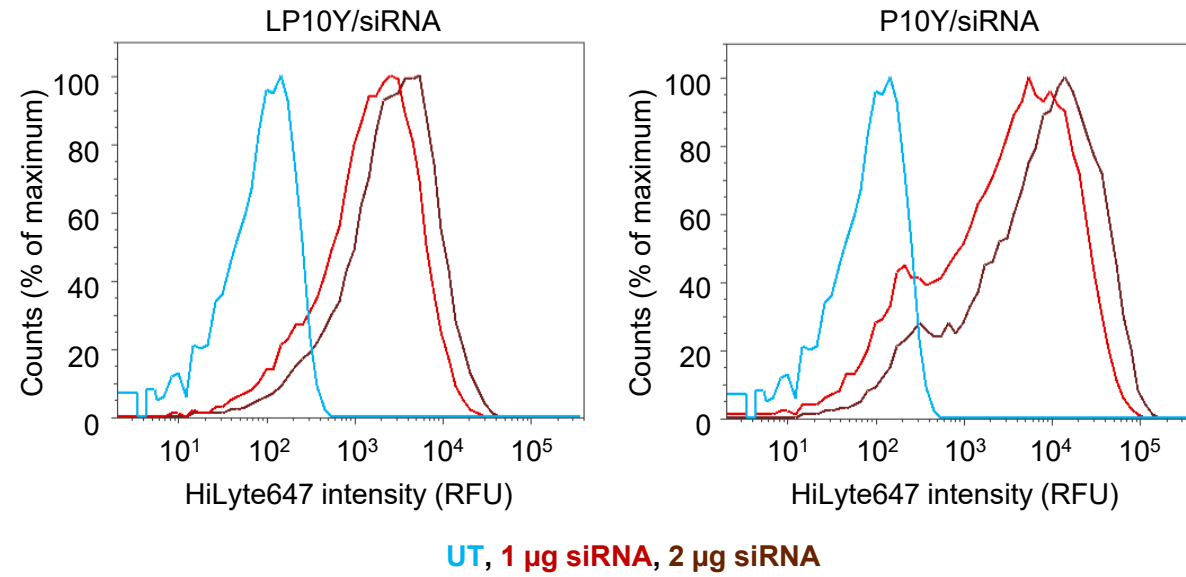

C

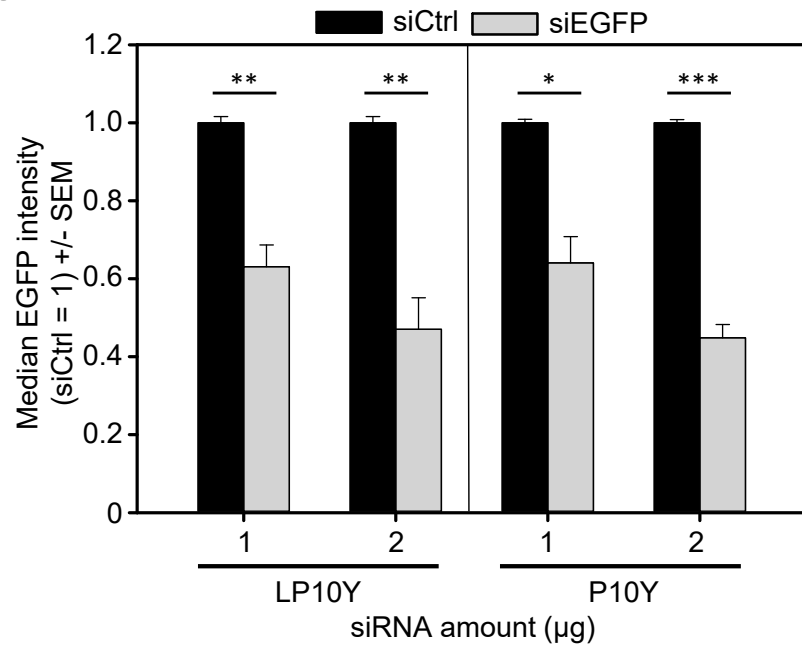

D

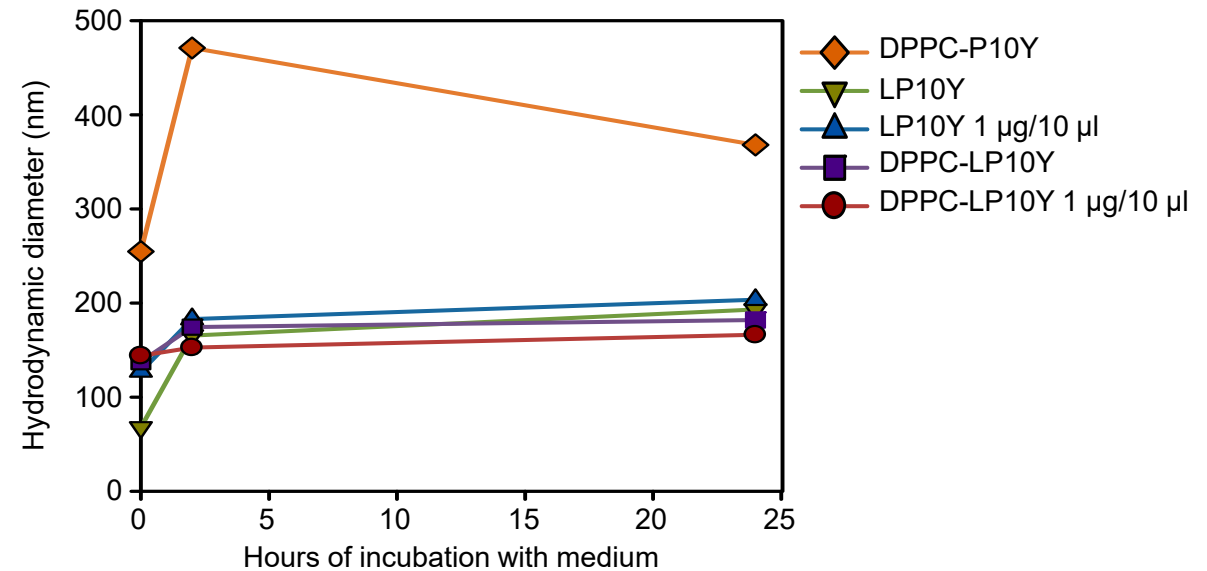

Figure S4

Supplement: Supplementary file 1 [file pharmaceutics-17-00339-s001.zip › Figure S4.pdf]
